# Delayed Climate Benefits and Toxicity Risks Could Hinder the Sustainable Deployment of Enhanced Weathering

**DOI:** 10.1021/acs.est.6c08993

**Published:** 2026-08-26

**Authors:** Selene Cobo, Gonzalo Guillén-Gosálbez

**Affiliations:** † Department of Chemical and Biomolecular Engineering, 16761 University of Cantabria, Santander 39005, Spain; ‡ Department of Chemistry and Applied Biosciences, Institute for Chemical and Bioengineering, ETH Zürich, Zürich 8093, Switzerland

**Keywords:** CO_2_ removal, negative emissions, enhanced weathering, greenhouse gas removal efficiency, toxicity, human health, ecosystem quality

## Abstract

Enhanced weathering has gained attention as a promising CO_2_ removal (CDR) practice, but previous life cycle analyses rely on theoretical assumptions that differ substantially from experimental observations and overlook the risks of heavy metal emissions. Here, we draw on data from existing empirical studies to conduct a prospective life cycle assessment of multiple enhanced weathering scenarios. Our results indicate that experimental weathering rates lead to delayed climate benefits, with a median 11 year lag between rock application and the onset of net CDR for inland scenarios starting in 2030. This carbon payback period results from the initial peak in greenhouse gas emissions associated with the rock supply chain and processing activities. Furthermore, our probabilistic analysis indicates that basalt-derived zinc emissions to agricultural soil could pose a greater toxicological risk than nickel release from dunite in the long term, potentially offsetting the health cobenefits of CDR and challenging the prevailing assumption that basalt is a safer feedstock–both on a per-mass-of-rock and a per-CDR basis. These findings suggest that the commercialization of CDR credits should be preceded by a thorough evaluation of near-term net climate benefits, and deployment should prioritize nonagricultural areas until the risks of food chain contamination are better understood.

## Graphical abstract

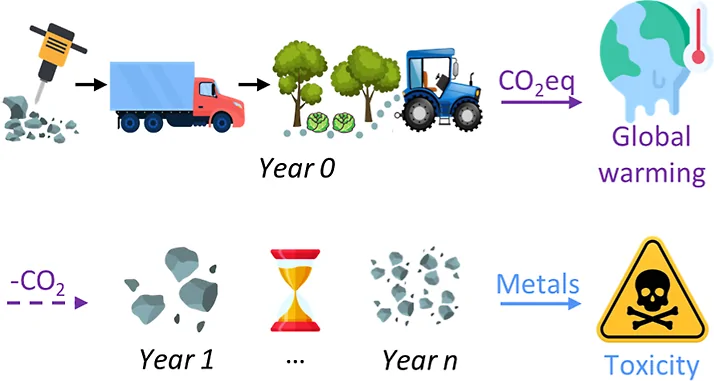

## Introduction

Solely relying on emissions reductions will most likely be insufficient to limit global warming to 2 °C above preindustrial levels. Scenarios assessed by the IPCC indicate that, in addition to deep and rapid decarbonization efforts, CO_2_ removal (CDR) from the atmosphere will most likely be required to offset residual emissions and reverse a temporary temperature overshoot.^1^ The timing of CDR deployment is therefore critical, and delays are expected to compound the technical and economic risks.^2^

To scale up CDR at the required pace, a portfolio of Negative Emissions Technologies or Practices (NETPs) spanning a range of maturity levels is currently being developed.^3^ Yet, previous studies have identified a range of sustainability trade-offs across CDR methods, most notably regarding health effects, planetary boundary transgressions, intensive resource demands, and the opportunity costs of energy allocation.^4–6^

The health and environmental implications of enhanced weathering remain under-researched relative to other NETPs. This CDR approach accelerates natural weathering processes by grinding rocks containing silicate minerals and spreading them over land surfaces. The rock materials react with CO_2_ in the presence of water (as illustrated by reaction R1 below, where Me represents a divalent metal) and drive the transfer of additional atmospheric CO_2_ to the water.^7^ Runoff transports the resulting alkalinity to the ocean, where the residence time of the sequestered dissolved inorganic carbon is on the order of 10^5^ years.^8–10^

The potential cobenefits of enhanced weathering–chiefly, the mitigation of soil and ocean acidification,^8,11,12^ improvement of soil structure and water retention,^13^ and increased soil fertility,^14^ which in turn augment biomass CDR^15^–render it attractive relative to other NETPs. Furthermore, enhanced weathering could present a thermodynamic advantage over other CDR methods; its energy demand–though highly scenario-specific and tied to variables such as grain size, transportation distances and rock type–has been estimated to be lower (<4 GJ/t CO_2_ ^16^) than that of alternative NETPs such as direct air carbon capture and storage (<9 GJ/t CO_2_ ^17,18^ ).

However, experimental studies mimicking field conditions remain limited and report weathering rates up to 2 orders of magnitude lower than previously estimated^13,19,20^ due to multifactorial constraints such as surface area, hydrology, biota and system feedbacks.^21^ Moreover, the CDR potential of this NETP is highly dependent on site-specific conditions (such as temperature, moisture, and acidity^22,23^ ), and part of the removed CO_2_ could be released back to the atmosphere via the secondary precipitation of mineral carbonates if the system reaches supersaturation (R2), or through the reaction of acidic species with the generated bicarbonate ions (R3).^24–26^ Notably, rock particles could also be weathered by a number of chemical species present in the soil^23^–namely surface-bound protons and exchangeable Al^3+^ inherent to soil characteristics, acids associated with plant and microbial activity, and nitric acid from fertilizer application–thereby reducing the amount of rock available for CDR.

R1 $$Me_{2}SiO_{4}+4CO_{2}+4H_{2}O\to 2Me^{2+}+4HCO_{3}^{-}+H_{4}SiO_{4}$$

R2 $$Me^{2+}+2HCO_{3}^{-}\to MeCO_{3}+CO_{2}+H_{2}O$$

R3 $$H^{+}+HCO_{3}^{-}\to CO_{2}+H_{2}O$$

In addition to these geochemical uncertainties, it has been suggested that rock weathering may not yield inorganic CDR within short time scales.^27^ Lastly, toxic heavy metals can also be released as the rock particles dissolve.^28–30^ While experimental studies with basalt have not raised concerns about dangerously high heavy metal concentrations,^31–35^ nickel levels above regulatory limits have been found when olivine is used.^36,37^

Previous Life Cycle Assessment (LCA) studies quantifying the environmental impacts of enhanced weathering overlook these issues. They rely on estimated CO_2_ sequestration potentials^38–43^ or theoretical weathering rates^44–47^ instead of experimental data. Furthermore, most of them disregard the risk of heavy metal leaching, with only one work^41^ assessing the impacts of nickel release following the application of olivine-rich rocks on coastal areas.

Despite all the uncertainties surrounding the performance of this NETP,^48,49^ there has been a surge in enhanced weathering start-ups selling CDR credits.^50,51^ Thus, ascertaining the implications of enhanced weathering for human health and the environment is critical to ensure that it can be responsibly deployed.^52^

Here, we investigate the greenhouse gas removal (GGR) efficiency of enhanced weathering and its potential impacts on human health and ecosystems. Our analysis–grounded in reported experimental results and a comprehensive accounting of unintended emissions–assesses how applying rock particles with different compositions across various environments would affect these outcomes.

### Scenarios

We model 14 scenarios involving the application of rock particles to coastal areas and inland croplands and forestlands, using weathering rates derived from theoretical^53^ and experimental studies (laboratory,^54–56^ mesocosm^32,36^ and field^31^), which were normalized to a reference temperature of 15 °C. Given the varying experimental and environmental conditions across studies (summarized in Table S3 of the Supporting Information), we aim to establish a representative range of performance indicators for enhanced weathering, rather than conducting a direct comparative analysis of individual studies.

Our life cycle inventories comprise mining operations, rock transportation, comminution, and spreading, as well as the emissions occurring as the rocks weather. We focus on two rock types: basalt and dunite. Although each presents a distinct set of metal concentrations (see Tables 1.1 and 1.2 of the Supporting Information)^57^, previous studies assume that basalt poses fewer risks than dunite because of its lower nickel and chromium content.^14,58,59^

In cropland scenarios, we consider the phosphorus released from rock particles to substitute for an equivalent amount of synthetic phosphate fertilizers, thereby reducing fertilizer inputs while keeping crop yields constant relative to the baseline without rock application. By contrast, we assume that forest scenarios receive no industrial fertilizers; therefore, increased phosphorus availability enhances biomass carbon uptake relative to the baseline. Notably, we do not consider fertilization effects in the coastal scenarios given the many uncertainties regarding the efficiency and permanence of the carbon sequestration.^60–62^ Further details on the scenarios’ characteristics and enhanced weathering models are in Section 3 of the Supporting Information.

Our models are aligned with climate change mitigation projections generated by the REMIND^63^ 3.5 integrated assessment model. We focus on scenario SSP2-PkBudg1000, which assumes a middle-of-the-road shared socio-economic pathway (SSP2)^64^ and a peak carbon budget of 1000 Gt CO_2_eq, limiting global warming to 1.7–2.0 °C by 2100. We also test the sensitivity of our results to alternative climate trajectories based on Nationally Determined Contributions (SSP2-NDC) and National Policy implementations (SSP1-NPi), which lead to a temperature increase of 2.0–2.5 °C above preindustrial levels.

## Methods

We conduct an attributional^65^ prospective^66^ LCA using Brightway 2.5 ^67^ and the premise^68^ 2.3.3 python library to adapt activities in the Ecoinvent^69^ 3.11 database to scenario projections from REMIND^63^ 3.5.

The functional unit is defined as the gross geochemical removal of 100 Mt CO_2_ in one year through enhanced weathering reactions–excluding additional CDR from biomass fertilization effects to ensure a consistent cross-scenario comparison. Embedding the temporal dimension in the definition of the functional unit is essential to reflect how weathering kinetics–and not merely intrinsic alkalinity–determine the mass of rock required to achieve a given CDR target within a specific time frame. The proposed functional unit represents 26% of the total CDR projected for 2030 in the selected reference mitigation scenario and falls within techno-economic constraints,^70,71^ as detailed in Section 3.3 of the Supporting Information.

We calculate the time-dependent GGR efficiency of enhanced weathering (GGRη_*t*,*y*_) as the ratio between the net amount of CO_2_eq removed (i.e., the difference between the atmospheric CO_2_ sequestered after reacting with silicate minerals and the CO_2_eq emitted throughout the rocks’ life cycle) and the total amount of CO_2_ removed from the atmosphere via enhanced weathering reactions *t* years after the rock particles are applied to the soil in year *y*–as shown in eq E1, where CDR_*s*,*t*,*y*_ and GHG_*s*,*t*,*y*_ represent the gross amount of substance *s* within the set *J* of greenhouse gases that is either removed or emitted in year *t* after the year *y* of deployment (assumed to be 2030 or 2050). The dynamic global warming potential (GWP_*s*,*t*_, estimated with Equations SE1–5 in the Supporting Information and based on^72^) reflects that CDR near the end of the 100 year time horizon prevents less warming than CDR at the beginning.

E1 $$GGR\eta_{t,y}=\frac{\sum_{t^{\prime }=0}^{t^{\prime }=t}\sum_{s\in J}\left(\left|CDR_{s=CO_{2},t^{\prime },y}\right|-GHG_{s,t^{\prime },y}\right)\cdot GWP_{s,t^{\prime }}}{\sum_{t^{\prime }=0}^{t^{\prime }=t}\left|CDR_{s=CO_{2},t^{\prime },y}\right|}\;\forall t\in T,y\in Y$$

We quantify damage to human health and ecosystems relative to a baseline without CDR using the hierarchist perspective of the ReCiPe 2016 method.^73^ Health impacts, aggregated over the time horizon, are expressed in Disability-Adjusted Life Years (DALYs), representing the years of healthy life lost due to disability or premature death. Ecosystem impacts are quantified as the local species loss aggregated over space and time, expressed in species·year.

We assume a 50% release rate for phosphorus and metals from annually weathered rocks in our base scenarios, while exploring the full range (0 to 100%) within our uncertainty analysis. We estimate the health and ecosystem impacts averted by phosphorus fertilization differently for each scenario location. In cropland scenarios, these cobenefits are linked to the avoided production of synthetic phosphate fertilizers, consistent with the avoided burden LCA method.^65^ In forest scenarios, prevented health and ecosystem impacts arise from the secondary CO_2_ sequestered by the phosphorus-induced biomass growth (1.6–6.7 kg C/kg P^74^).

CDR prevents health and ecosystem impacts linked to the averted rise in global temperatures.^5,6^ These cobenefits (alongside those related to the substitution of industrial fertilizers in the cropland scenarios) may be–partially or entirely–offset by the health and ecosystem impacts associated with the resources used and pollutants emitted throughout the enhanced weathering systems and their underlying supply chains. Hence, we define net health and ecosystem damage as the balance between the impacts generated and avoided by CDR (equations SE6 and SE7 in the Supporting Information); positive values imply that undesired impacts outweigh cobenefits, and negative values indicate that prevented impacts are greater than detrimental side-effects.

We use eq E2 to calculate MT_*n*,__ec_^H^, i.e., the human toxicity effects of metal emissions to environmental compartment *ec* (seawater, agricultural soil, or forest soil, depending on the scenario location) due to the annual weathering of rock *n* (DALY/year). Here, RM_*n*_^*A*^ represents the mass of rock *n* that weathers annually (kg/year), *C*_*s*,*n*_ is the concentration of substance *s* within the set *ME* of metals in rock *n* (kg/kg), *f*_*s*,ec_ denotes the fraction of metal *s* emitted to environmental compartment *ec*, and TF_s,__ec_^C^ and TF_s,__ec_^NC^ are the carcinogenic and noncarcinogenic toxicity factors for metal *s*, respectively, provided by the ReCiPe 2016 method^73^ (DALY/kg). Toxicity factors account for the environmental persistence and bioavailability of the emitted metals. Notably, long-term metal intake is primarily driven by dietary ingestion,^75^ as detailed in Section 2.2 of the Supporting Information.

$$MT_{n,ec}^{H}=\underset{s\in ME}{\sum }RM_{n}^{A}\;\cdot \;C_{s,n}\;\cdot \;f_{s,ec}\;\cdot \;\left(TF_{s,ec}^{C}+TF_{s,ec}^{NC}\right)\;\forall n\in N,ec\in EC$$ E2

To estimate the probability distributions of the human toxicity impacts associated with metal emissions, we perform 10,000 Monte Carlo simulations in which RM_*n*_^*A*^, *C*_*s*,*n*_ and *f*_*s*,ec_ are sampled from uniform distributions, which avoids introducing unsupported assumptions by treating all values within the plausible range as equally likely. *f*_*s*,ec_ changes between 0 and 1 to account for metal mobilization driven by possible fluctuations in soil pH and redox potential,^76^ RM_*n*_^*A*^ varies between the minimum and maximum values estimated across the inland scenarios modeled for each rock type, and *C*_*s*,*n*_ takes values within the ranges reported in the literature.^36,77–79^

Figure 1 outlines the methodological sequence followed in this study. Additional data and assumptions underpinning our models are described in Section 3 of the Supporting Information. Life cycle inventories are compiled in the Supporting Information.^57^

**Figure 1:**
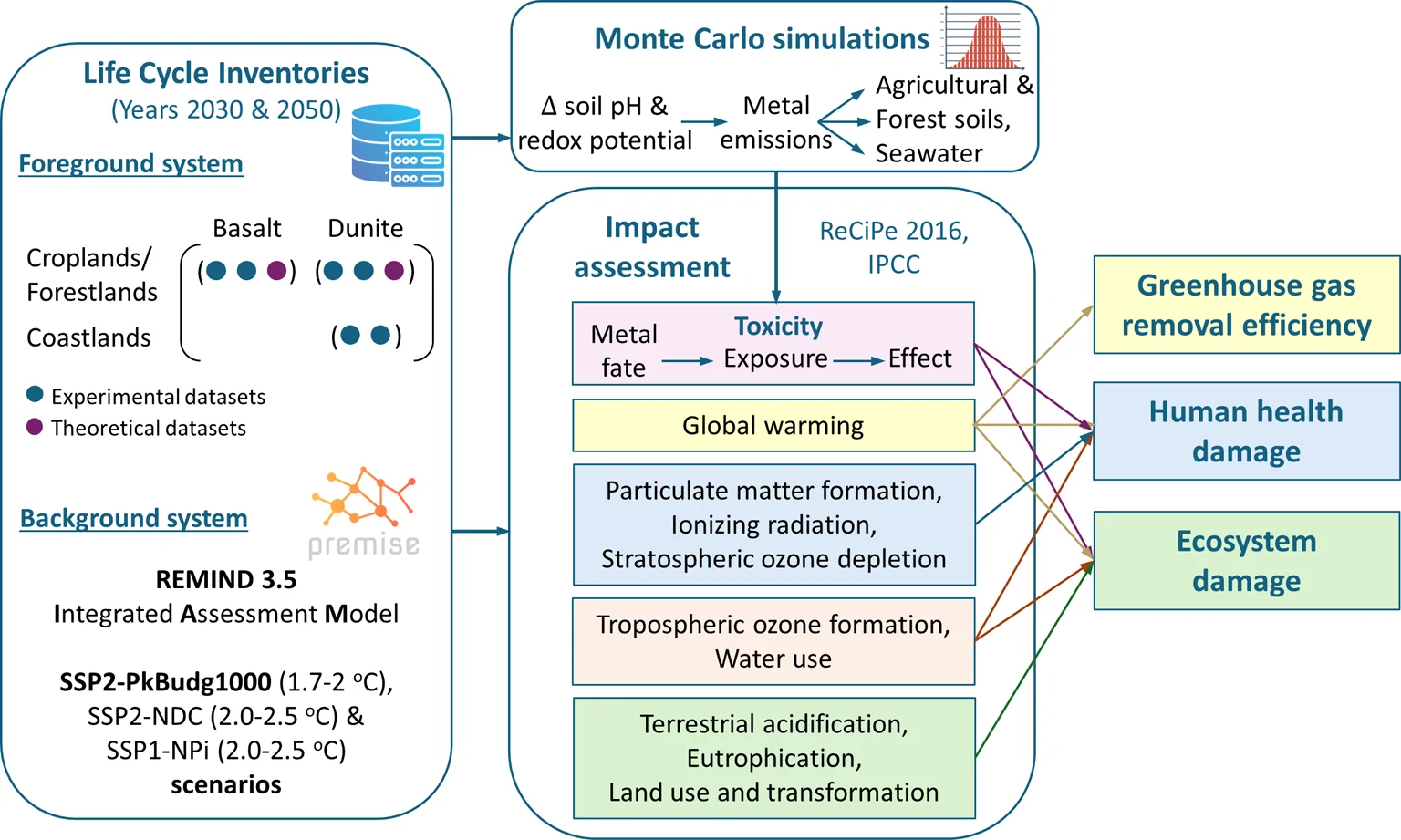
Data flow and integrated methodological framework used for the prospective life cycle assessment of the enhanced weathering scenarios.

## Results

### Delayed Climate Benefits

Figure 2 illustrates how the GGR efficiencies of our scenarios improve as the rocks progressively weather over time, increasing CDR. GGR efficiencies are dictated by the weathering rates, with scenarios considering theoretical kinetic data achieving better outcomes than those based on the slower rates reported in experimental studies. As expected, in the theoretical scenarios assuming identical particle sizes and soil conditions, dunite outperforms basalt because of its higher alkalinity content, which implies that less rock is required to achieve the same CDR (Supporting Figure S2). We also observe lower GGR efficiencies for scenarios starting in 2030 (Figure 2a) relative to 2050 (Figure 2b), as the gradual adoption of decarbonization measures increasingly reduces the carbon footprint of the activities involved in the rock supply chains.

**Figure 2:**
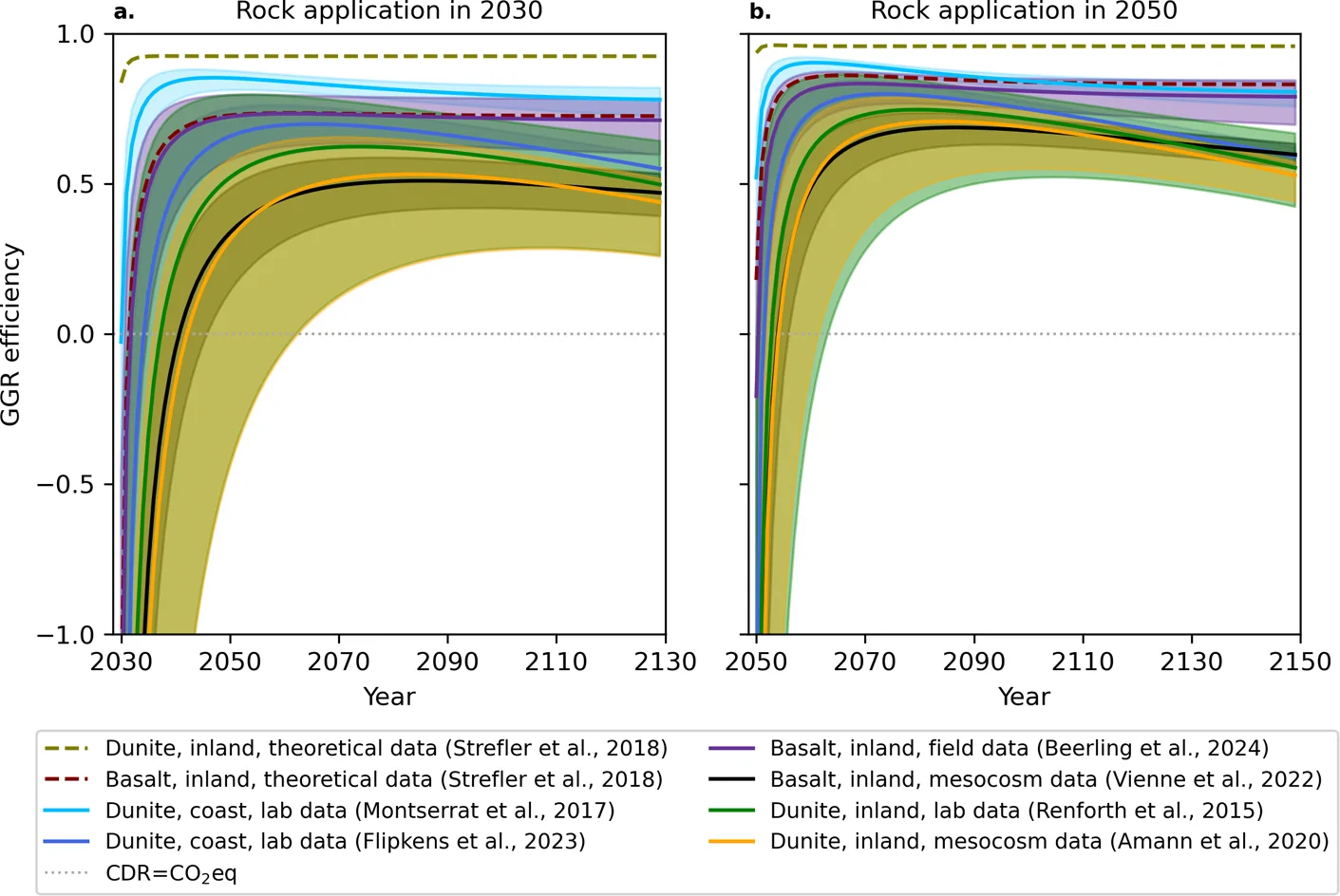
Evolution of greenhouse gas removal (GGR) efficiencies over 100 years, considering experimental^31,32,36,54–56^ and theoretical^53^ data for the application of dunite and basalt on coastal and inland environments. The shaded areas represent the uncertainty in the results. (a) Single rock application in 2030. (b) Single rock application in 2050.

In the inland scenarios modeled after experimental results, initial CDR is insufficient to offset greenhouse gas emissions associated with rock extraction, transportation, and processing, which are assumed to occur in the year that the rock particles are applied to the soil. Indeed, these experimental scenarios only attain positive GGR efficiencies (removing more CO_2_eq than is emitted) 2–14 years after the rock application (central values), with the exact timing depending on the scenario and year of deployment. If the least favorable parameters are considered, it takes up to 34 years for rock particles applied in 2030 to achieve net CDR.

While the inland scenarios based on experimental data from^32,36,56^ achieve modest GGR efficiencies by the end of the 100 year time horizon (0.44–0.60, central values), the scenario considering data from a field trial with basalt^31^ yields higher GGR efficiencies (0.71–0.79), similar to those of the scenario relying on theoretical basalt weathering rates. Indeed, this is the only scenario based on experimental data that achieves complete weathering within 100 years (Supporting Figure S3 and Table S4). This discrepancy could be attributed to differences in the methods used to quantify weathering rates; studies^32,36,56^ determined these rates based on alkalinity concentrations in the effluent of the experimental set-ups, whereas^31^ relies on the residual cation content measured in soils. This soil-based technique assumes that all cation losses lead to CDR, overlooking cation uptake by plants and formation of secondary minerals.^51^

The coastal enhanced weathering scenario based on^54^ does not show a significant delay in the climate benefits, achieving GGR efficiencies of 0.73–0.84 at the end of the 100 year time horizon. By contrast, the coastal scenario modeled after^55^ attains worse results (positive GGR efficiencies 2–9 years after the rock application, reaching values of 0.50–0.62 after 100 years), although it outperforms the inland scenarios using the same rock type. This could be in part due to the advantageous conditions of the simulated coastal environments, where dunite particles were directly subjected to agitation in seawater, omitting the initial transport phase.

The theoretical scenarios attain the highest GGR efficiencies for each rock type (0.73–0.96 at the end of the studied period). Notably, GGR efficiencies in the dunite theoretical scenarios stabilize shortly after the initial peak in emissions because weathering happens quickly. Here, 100% of dunite grains have weathered 14 years after being applied, while basalt reaches this point 70 years after the rock application (Supporting Figure S3 and Table S4). Conversely, the GGR efficiency curves for the scenarios based on experimental data present a slight decline after reaching their maximum values. The reason is that the denominator of the GGR efficiency (eq E1) grows faster than the numerator because the dynamic global warming potential progressively decreases over time and reaches 0 at the end of the time horizon–at which point additional greenhouse gas emissions no longer contribute to warming within the assessed period.

Results for enhanced weathering scenarios aligned with alternative climate change mitigation pathways (Supporting Figure S4) follow the same patterns but exhibit variability in GGR efficiencies relative to SSP2-PkBudg1000 (Figure 1). Specifically, efficiencies decrease by up to 20% at the end of the studied period under the SSP1-NPi scenario, whereas they increase by up to 18% under the SSP2-NDC pathway. The latter enforces more aggressive decarbonization targets, thereby reducing the upstream greenhouse gas emissions associated with rock processing and transport.

### Health and Ecosystem Cobenefits Offset by Life Cycle Emissions

Here we evaluate how human health and ecosystem quality are affected by the gross removal of 100 Mt CO_2_ in 2030 (Figure 3a) and 2050 (Figure 3b). We assume a regime of consecutive annual deployments, where rock particles are initially applied in 2030, and the amount of rock weathered the previous year must be subsequently applied to maintain a constant CDR rate (details in Section 3.1 of the Supporting Information). Hence, the bulk of the rock processing activities occur in 2030, which is reflected in the worse environmental performance of the analyzed scenarios in the initial year.

**Figure 3:**
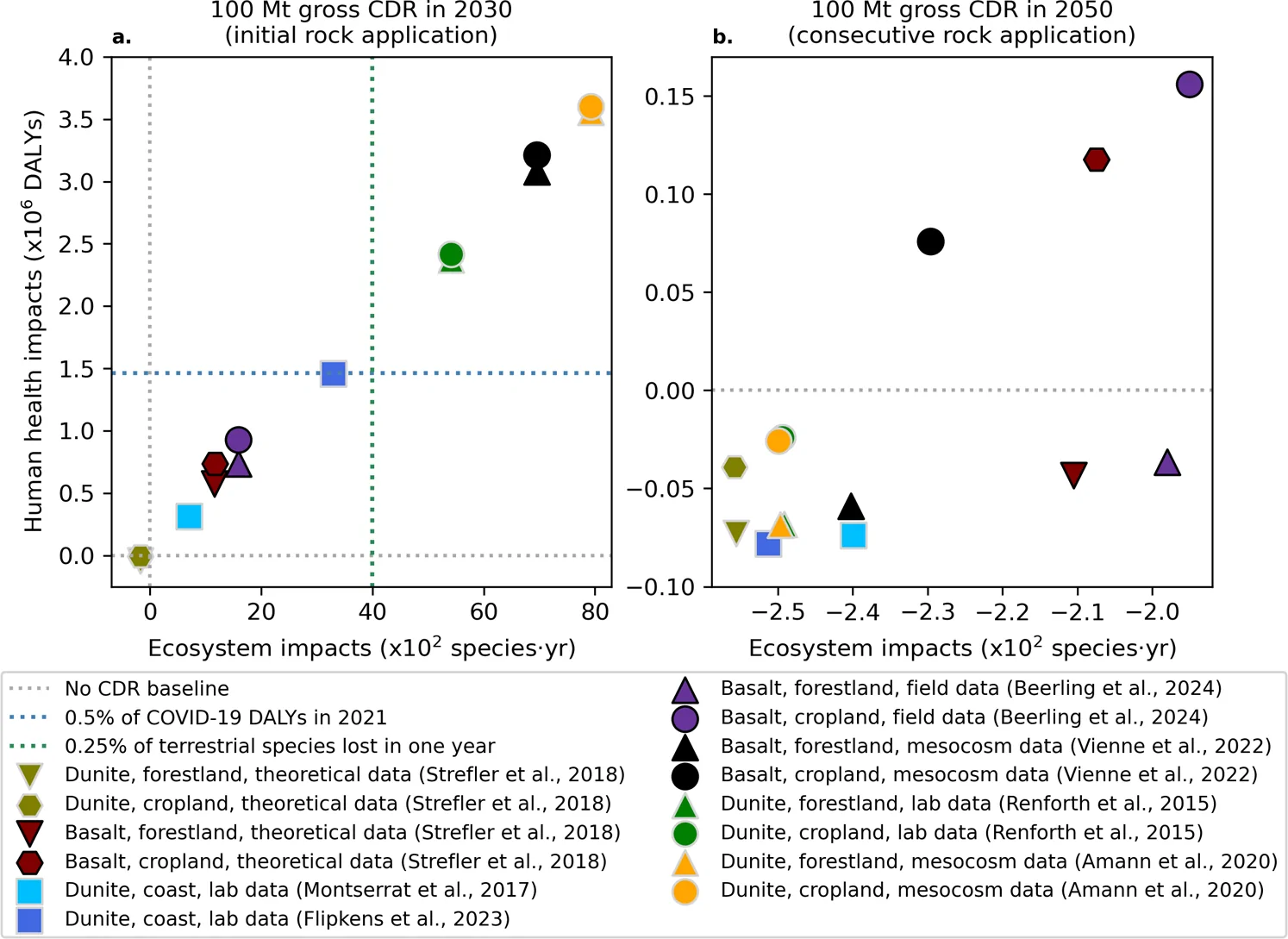
Human health and ecosystem impacts of removing 100 Mt CO_2_ (gross) in 2030 and 2050, considering experimental^31,32,36,54–56^ and theoretical^53^ data for the application of dunite and basalt on coastal and inland (forestlands and croplands) environments. (a) Rock application in 2030 for the first time. (b) Consecutive rock application in 2050.

In 2030, health and ecosystem impacts correlate with GGR efficiencies, with the most efficient scenarios yielding the lowest overall damage. Inland scenarios based on mesocosm and laboratory experiments^32,36,56^ cause the most harmful effects during the initial year of deployment (2.4 · 10^6^–3.6 × 10^6^ DALYs and 5.4 · 10^3^–7.9 × 10^3^ species·year), which are comparable in magnitude to 0.8–1.2% of the damage caused by COVID-19 in 2021 ^80^ and to the loss of 0.3–0.5% of the world’s terrestrial species in one year.^73^ Notably, only the theoretical dunite scenarios avert net health and ecosystem damage during the first year of rock application. In these cases, the CDR cobenefits are enough to counterbalance the undesired consequences of resource use and pollutant emissions on human health and ecosystems.

Conversely, in 2050 all scenarios prevent net ecosystem impacts (between −2.6 × 10^2^ and −2.0 × 10^2^ species·year), with the basalt scenarios exhibiting the worst outcomes. Only the three scenarios considering basalt spreading on croplands generate net health damage (≤1.6 × 10^5^ DALYs). The poorer performance of basalt scenarios in 2050 can partially be explained by basalt’s lower alkalinity content, which entails that larger amounts of rock must be weathered to achieve the same annual CDR as dunite, leading to higher upstream impacts. This scenario ranking deviates from that of 2030, where the best-performing inland scenarios based on experimental results corresponds to the basalt data set with the highest temperature-normalized alkalinity dissolution rate (derived from^31^) across scenarios, whereas the dunite scenario with the lowest dissolution rate (data from^36^) is the most damaging.

Equations SE12–15 in the Supporting Information explain these divergent trends. While the amount of rock that must be applied in the initial year of deployment is inversely proportional to the alkalinity dissolution rates, in subsequent years the amount of rock that has been weathered–and must therefore be reapplied to maintain a constant annual CDR–is inversely proportional to the rock’s alkalinity concentration. We note that health and ecosystem impacts projected for alternative mitigation pathways (Supporting Figure S5) follow the same trends, varying between −14% and +7% compared to the climate mitigation scenario considered here.

Figure 4 displays the disaggregated health impacts of two representative inland scenarios based on data from mesocosm experiments for basalt^32^ and dunite,^36^ with results tied to the unique experimental conditions of each study. In the initial year of rock application (2030), >99% of the undesired health impacts in both scenarios arise from the greenhouse gases, particulate matter and toxic substances emitted throughout the rock supply chains and processing activities–over 64% of which are linked to rock transportation (road transport distances of 180–250 km^81^). In the selected scenarios, the health impacts of removing 100 gross Mt CO_2_ with basalt (Figure 4a) and dunite (Figure 4d) in 2030 amount to 3.1 × 10^6^ (2.2 × 10^6^, 4.4 × 10^6^) and 3.6 × 10^6^ (1.9 × 10^6^, 8.4 × 10^6^) DALYs, respectively.

**Figure 4:**
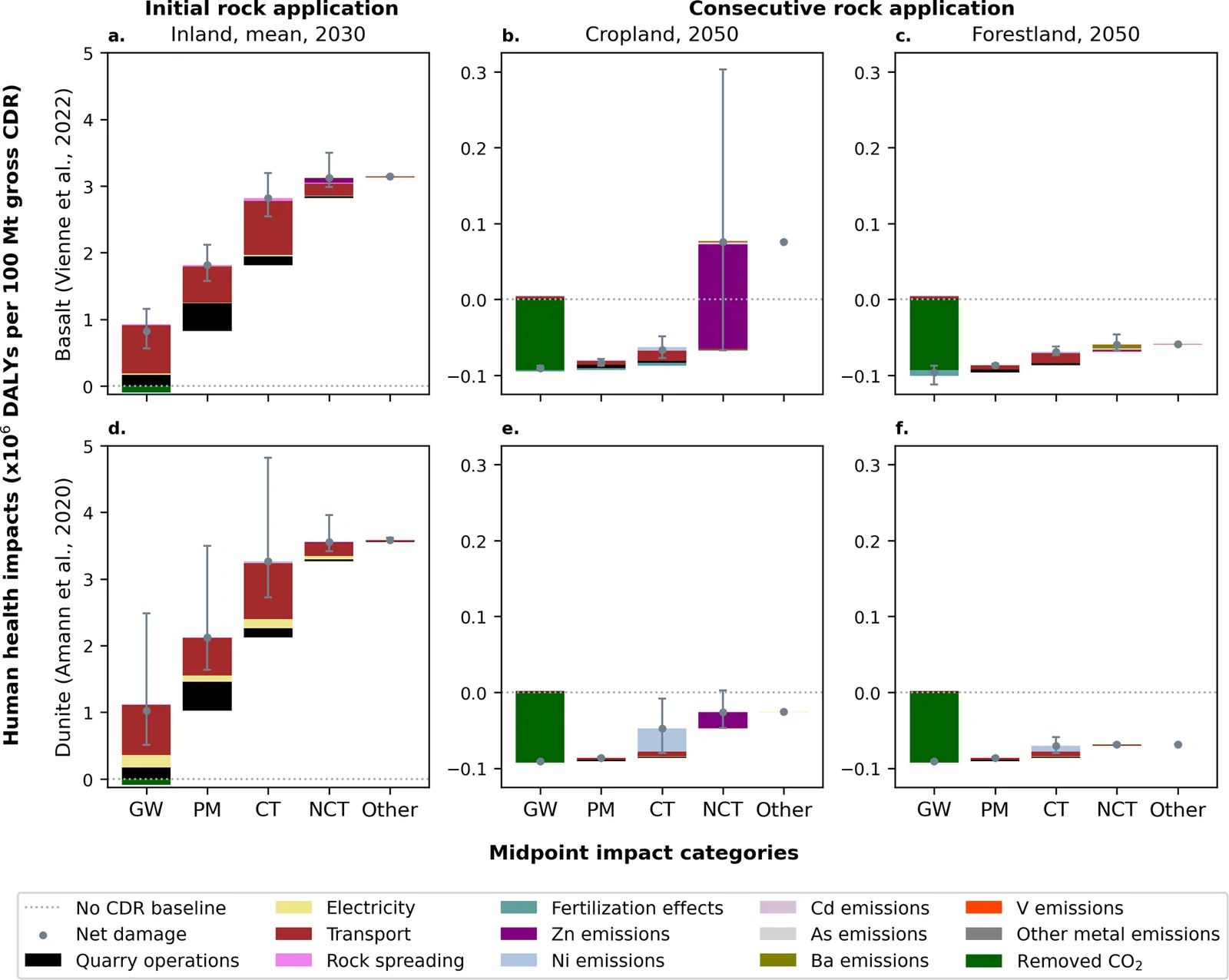
Human health impacts of removing 100 Mt CO_2_ (gross) in 2030 and 2050, considering initial rock application in 2030 and consecutive applications in subsequent years. Data from.^32,36^ Error bars represent the uncertainty in the results. Impacts disaggregated by the contribution of (i) unit processes and direct emissions, and (ii) environmental mechanisms: global warming (GW), fine particulate matter formation (PM), carcinogenic toxicity (CT), noncarcinogenic toxicity (NCT), and others (tropospheric ozone formation, stratospheric ozone depletion, ionizing radiation, and water consumption). (a) Basalt application to cropland/forestland (mean), 2030. (b) Basalt application to cropland, 2050. (c) Basalt application to forestland, 2050. (d) Dunite application to cropland/forestland (mean), 2030. (e) Dunite application to cropland, 2050. (f) Dunite application to forestland, 2050.

After the initial year of rock application–when most of the rocks are mobilized–health outcomes are primarily determined by the potential metal release and the CDR cobenefits. Our results show that the metals contained in basalt could lead to greater health problems than those in dunite. Zinc emissions from basalt grains applied to croplands can cause significant noncarcinogenic toxicity effects, leading to net health damage in 2050:7.6 × 10^4^ (−8.4 × 10^4^, 3.3 × 10^5^) DALYs due to the removal of 100 gross Mt CO_2_ (Figure 4b). Dunite can also emit zinc in lower amounts, but nickel–conducive to carcinogenic toxicity impacts–poses a greater health risk with this rock. Nevertheless, the health cobenefits of CDR could offset the toxicity effects of metal release from dunite, preventing net damage; the health impacts of removing 100 Mt CO_2_ by applying dunite to croplands in 2050 amount to −2.6 × 10^4^ (−8.0 × 10^4^, 4.4 × 10^4^) DALYs (Figure 4e).

Spreading rock grains in forests is less hazardous to humans because of the lower risk of metals entering the food supply chain. The gross removal of 100 Mt CO_2_ in 2050 with basalt applied to forestland can also avert net health damage, i.e., −5.9 × 10^4^ (−8.9 × 10^4^, −2.8 × 10^4^) DALYs (Figure 4c), similar to the net impacts prevented with dunite: −6.9 × 10^4^ (−8.0 × 10^4^, −5.4 × 10^4^) DALYs (Figure 4f). As Figure 4 shows, the relative contribution of metal emissions to overall health impacts in the cropland scenario deploying basalt is significantly higher than in alternative rock–location combinations. This explains the wider gap in health impacts between basalt applied to cropland versus forestland (Figure 3), whereas this location-dependent difference is much smaller for dunite. However, if the nickel content in dunite is released into coastal environments at high rates such as those reported in,^54^ the associated toxicity effects could offset as much as 91% of the health cobenefits of CDR in the initial year of deployment (Supporting Figure S6).

As Supporting Figure S7 shows, the damage to ecosystem quality due to metal release is negligible in the experimental inland scenarios, where >66% of detrimental ecosystem impacts are attributed to global warming and land use associated with rock transportation and quarry operations. The difference in ecosystem damage between croplands and forestlands is driven primarily by the distinct fertilization effects of phosphorus release in these environments. Conversely, the ecotoxicity effects of nickel emissions could play a more important role in marine environments, offsetting up to 87% of the CDR ecosystem cobenefits of the coastal enhanced weathering scenarios in 2030 (Supporting Figure S6).

Extrapolating robust conclusions on the relative performance of dunite and basalt by comparing individual studies is challenging due to the diverse conditions under which experiments were conducted, as well as the different techniques used to estimate weathering rates.

While dunite removes more CO_2_ per unit mass after complete weathering because of its higher alkalinity content, some scenarios require less basalt to meet the CDR target due to the combination of (1) the faster weathering kinetics reported, and (2) the short one-year weathering period defined by our functional unit. Hence, those scenarios present lower health and ecosystem impacts linked to the rock processing activities.

Specifically, the initial mass of basalt that must be applied based on data from^31^ is lower than the dunite mass derived from.^36,55,56^ Similarly, the initial amount of basalt estimated using results from^32^ is smaller than the amount of dunite derived from data reported in^36^ (rock requirements in Supporting Information),^57^ which is reflected in the scenario ranking depicted in Figure 3a.

To remove the confounding effect of varying rock quantities across scenarios and better illustrate the rock’s performance, we show in Supporting Figure S8 the health and ecosystem impacts resulting from the initial application of 1 Mt of rock particles in 2030 and the reapplication of the scenario-specific rock amounts required in 2050 to maintain each scenario’s annual CDR rate–i.e., each year begins with 1 Mt of unweathered rock, consisting of particles remaining from previous years plus new particles added to replace those weathered during the preceding year.

Supporting Figure S8 proves that, on an equivalent mass basis, dunite tends to outperform basalt in terms of health and ecosystem impacts, with six of the eight best-performing scenarios in 2030 deploying dunite. However, rock type is not a perfect predictor of relative scenario performance; the disaggregated health effects of two representative scenarios (Supporting Figure S9) reveal that, when considering rock mass as the functional unit, differences in health impacts are mainly driven by the electricity used for particle grinding (dependent on particle size) in the initial year of rock application, and by the toxicity of metal emissions (inherent to the rock composition) in subsequent years.

We further investigate how rock composition influences health impacts by comparing the toxicity potential of both rocks. We define this metric as the theoretical maximum human toxicity effects from metal release per unit mass of rock weathered within one year, assuming all metals leach into the soil.

Supporting Figure S10 shows that, when rock particles are applied to croplands, the toxicity potential of basalt is 34% higher than that of dunite because of basalt’s greater zinc content combined with the high toxicity factor of zinc emissions to agricultural soils. Conversely, in forest environments, nickel emissions drive the toxicity potential of dunite to twice that of basalt, given its substantially larger nickel content and the similar toxicity factors of zinc and nickel in forest soils.

Nevertheless, the toxicity potentials of basalt and dunite applied to croplands are 17 and 6 times greater, respectively, than when applied to forestlands due to the higher risk of human exposure via food intake, highlighting the need for greater scrutiny in agricultural settings.

### Basalt Entails Higher Long-Term Toxicity Risks in Agricultural Soils

Potential metal release to agricultural soils introduces significant uncertainty into the human toxicity assessment (Figure 4). To address this knowledge gap in the long term fate of metals, we conduct Monte Carlo simulations characterizing the probability distribution of toxicity impacts associated with metal emissions from rock particles applied to croplands (Figure 5).

**Figure 5:**
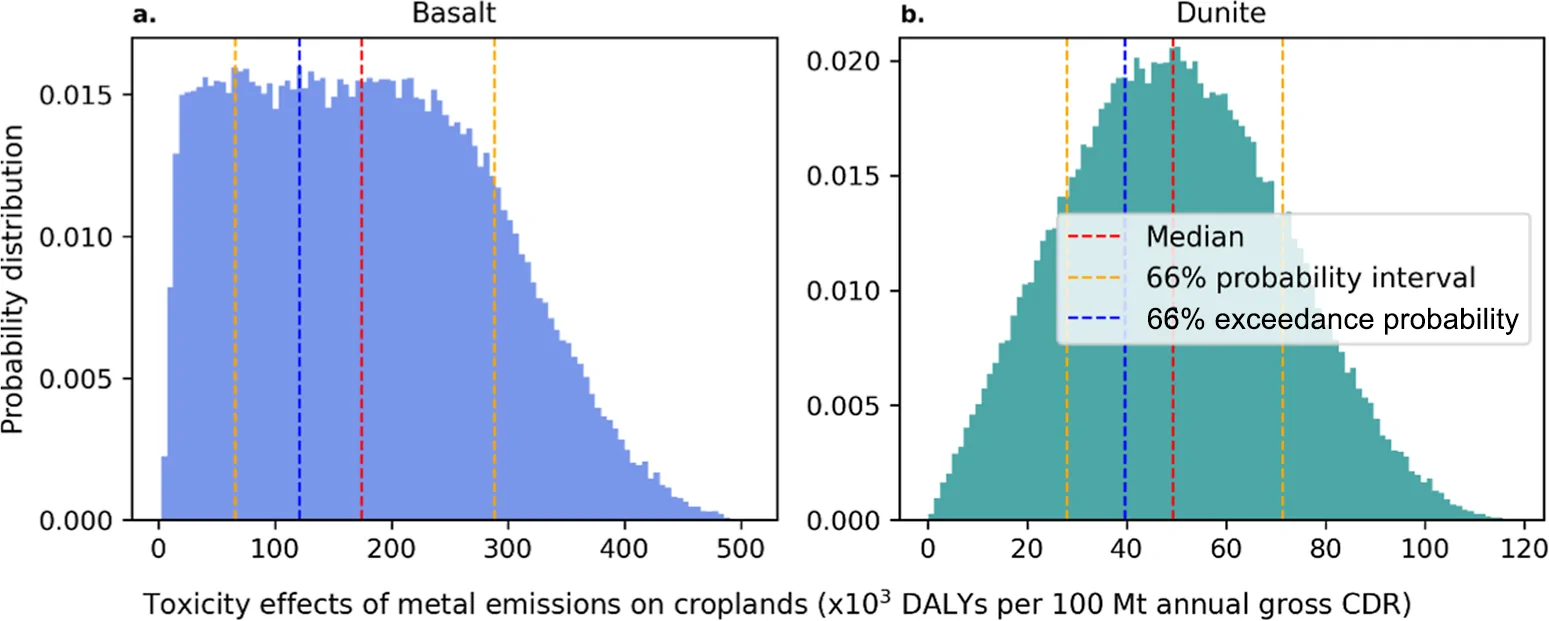
Probability distributions of toxicity-driven human health impacts from metal emissions to agricultural soil, calculated with the hierarchist perspective of the ReCiPe 2016 method. Data represent Monte Carlo simulation results for the annual removal of 100 Mt CO_2_ (gross) using (a) Basalt. (b) Dunite.

We find that deploying basalt and dunite to remove 100 Mt gross CO_2_ entails a 66% likelihood that associated metal emissions would lead to health impacts exceeding 1.2 × 10^5^ and 4.0 × 10^4^ DALYs, respectively. The median health toxicity effects linked to metal release from basalt are 1.7 × 10^5^ DALYs (66% likelihood: 6.6 · 10^4^–2.9 × 10^5^ DALYs, Figure 5a), 3.5 times the impacts associated with dunite, i.e., 4.9 × 10^4^ DALYs (66% likelihood: 2.8 · 10^4^–7.1 × 10^4^ DALYs, Figure 5b). Although the toxicity effects of basalt are more dispersed than those of dunite (coefficients of variation: 0.58 and 0.43, respectively), the low overlap between the likely toxicity ranges of the two rocks underscores the higher risks of basalt in agricultural areas.

The probability distribution of basalt’s toxicity effects is virtually flat shaped across a wide range of values, consistent with the underlying uniform distributions. Since uniformly distributed variables are combined multiplicatively (eq E2), combinations of high values reinforce one another, producing large outcomes. This amplification stretches the right tail of the distribution and generates the observed positive skewness.

By contrast, the dunite distribution follows a highly symmetric and unimodal shape, indicating that outcomes near the median are significantly more likely than extreme values. Although the metal emissions from dunite follow uniform distributions (Supporting Figure S11), its aggregate toxicity effects are dominated by two metals–nickel and zinc–with different characterization factors, whereas basalt’s toxicity is mainly driven by zinc (Figure 4). Hence, the combination of distinct toxicity contributors in dunite leads to a probability distribution converging toward a Gaussian shape, consistent with the central limit theorem.^82^

The metal-induced toxicity effects depicted in Figure 5 are calculated using characterization factors that reflect impact mechanisms based on current scientific consensus and a 100 year time horizon. Nevertheless, metal toxicity impacts are highly dependent on the selected time frame.^83^ As shown in Supporting Figure S12, alternative value choices–regarding time horizons and acceptable degrees of scientific evidence–yield different results.

Notably, if a precautionary framework based on an infinite time horizon is adopted, the toxicity impacts of both rocks increase by 1 order of magnitude. By contrast, under a 20 year techno-optimiztic perspective, the human toxicity impacts of dunite and basalt are one and 3 orders of magnitude lower than in the consensus-based perspective, respectively.

Under the optimiztic perspective, basalt is preferable to dunite. The reason is that nickel poses a greater risk than zinc via inhalation and drinking water consumption–the only human exposure routes considered in the short term. Conversely, over longer timelines where exposure via food intake is accounted for, the bioaccumulation of zinc in food products causes basalt’s integrated toxicity score to overtake that of dunite.

## Discussion

Our analysis reveals the potential risks of relying on enhanced weathering for immediate climate benefits. Notably, inland scenarios based on experimental weathering rates show a median 11 year lag between the initial rock application in 2030 and the onset of net CDR. Although the estimated delay remains sensitive to the uncertainties inherent in the weathering rate measurement techniques used in the considered experimental studies, our results suggest that representative weathering rates may be insufficient to offset the peak in greenhouse gas emissions associated with the rock supply chain and processing activities within climate-relevant time frames. Such delays in net CDR imply that enhanced weathering should not be viewed as an emergency intervention to rapidly shorten the duration of a temporary temperature overshoot, as the initial carbon debt could lead to additional warming in the near term.

While these kinetic barriers could be bridged–via optimal grain size,^84^ site selection,^85^ irrigation,^86^ pH management,^85,87^ or the integration of biological accelerators such as mycorrhizal fungi^88^–the potential health risks associated with metal leaching remain a concern. Our probabilistic analysis reveals that the long-term toxicity effects of basalt applied to croplands–primarily driven by zinc emissions–are greater than those of the dunite, both per unit mass of rock and per unit of achieved CDR. Thus, these findings challenge the notion that basalt is safer because of its lower nickel content.

Even if new research suggests that harmful metals are largely retained in the soil over meaningful time scales, their eventual release remains sensitive to future perturbations in the soil pH and redox conditions.^76^ These changes are driven by an interplay of physicochemical and biological processes^76,89^ that are difficult to monitor and regulate at scale–such as atmospheric deposition and the resulting soil acidification, or soil respiration, which alters CO_2_ and oxygen levels in pore water. Consequently, it is essential to include potential metal emissions in life cycle and risk assessments. Future studies should investigate potential mixed-metal “cocktail effects” to determine whether concurrent metal releases compound cumulative toxicity.^90^

Despite these uncertainties, the likelihood of trace-metal mobilization triggered by redox shifts could be reduced by avoiding areas with low pH buffering capacity^91^ and poorly drained soils.^92^ However, given the complexity of soil biogeochemistry and based on these preliminary results, it may be advisible to adopt a precautionary approach by prioritizing nonagricultural areas for enhanced weathering trials. This could mitigate the projected risk of toxic heavy metals entering the food supply chain, thereby minimizing the potential for human exposure.

Our findings highlight that a robust monitoring, reporting, and verification framework, underpinned by field-scale evidence, remains essential to safeguard the integrity of carbon markets. Until kinetic lags and heavy metal dynamics are better understood, the sale of CDR credits from enhanced weathering may be premature. In the interim, investments should target pilot deployments designed to resolve these uncertainties and lay the scientific foundation for a safe and sustainable CDR industry.

## Supplementary Material



## Notes

The Supporting Information comprises additional details on the methodology, models, assumptions, limitations, and results described in the main manuscript. The Supporting Information^57^ (10.5281/zenodo.19073284) includes the rock composition, kinetic parameters, and enhanced weathering life cycle inventories (PDF)
