# Supplementary material for: Delayed Climate Benefits and Toxicity Risks Could Hinder the Sustainable Deployment of Enhanced Weathering
Source: Environ Sci Technol. 2026 Aug 26;60(35):24676–86. doi: 10.1021/acs.est.6c08993 (PMC13564415; doi:10.1021/acs.est.6c08993)
Supplement: Supplementary file 1 [file es6c08993_si_001.pdf]

Delayed climate benefits and toxicity risks could hinder the sustainable deployment of enhanced weathering

Supporting Information

Selene Cobo[1]* and Gonzalo Guillén-Gosálbez[2]

[1]Department of Chemical and Biomolecular Engineering, University of Cantabria, Spain

[2]Department of Chemistry and Applied Biosciences, Institute for Chemical and Bioengineering, ETH Zürich, Switzerland.

*cobos@unican.es

This document provides additional information on the methodology, models, assumptions, limitations, and results described in the main manuscript.

Table of contents

1. Nomenclature 3
2. Methods 6
   2.1 Greenhouse gas removal efficiency 6
   2.2 Health and ecosystem impacts 7
3. Enhanced weathering models 10
   3.1 Equations and variables 10
   3.2 Scenario parameters 12
   3.3 Main assumptions and limitations 15
4. Supporting results 17
5. References 28

1. Nomenclature

We define these main sets:
D ≔ {*d* | *d* ∈ Enhanced weathering datasets}
E ≔ {*e* | *e* ∈ Endpoint impact categories}
EC ≔ {*ec* | *ec* ∈ Environmental compartments}
L ≔ {*l* | *l* ∈ Locations}
M ≔ {*m* | *m* ∈ Midpoint impact categories}
N ≔ {*n* | *n* ∈ Rock types}
S ≔ {*s* | *s* ∈ Elementary flows}
T ≔ {*t* | *t* ∈ Number of years after the rock application}
T^P ≔ {t^p | t^p ∈ Number of years after pulse emission}
Y ≔ {*y* | *y* ∈ Years of rock application}

We also define the following subsets:
J ≔ {*s* | *s* Greenhouse gases}
ME ≔ {*s* | *s* Metals}

Table S1. Parameters and variables used to estimate the greenhouse gas removal efficiency and health and ecosystem impacts.

Symbol	Description	Unit
$\boldsymbol{a^0}$	CO_2 fraction that remains in the atmosphere for an infinite number of years after a pulse emission	–
$\boldsymbol{a^g}$	CO_2 fraction transferred to carbon sink *g* an infinite number of years after a pulse emission.	–
$\boldsymbol{AGWP_{CO_2}^{TH}}$	Absolute global warming potential of CO_2 over a 100-year period	W·year/m^2/kg
$\boldsymbol{AGWP_{s,t}^{TH'}}$	Absolute global warming potential of greenhouse gas *s* emitted or removed in year *t* and quantified over period TH'	W·year/m^2/kg
$\boldsymbol{C_{s,t^p}}$	Fraction of greenhouse gas *s* that remains in the atmosphere t^p years after being emitted	–
$\boldsymbol{CDR_{s,ec,t,y}}$	Gross amount of CO_2 removed from the atmosphere in year *t* after deployment in year *y*	kg/functional unit[a]
$\boldsymbol{CF_{m,s,ec,t}^{MID}}$	Characterization factor quantifying the impacts of elementary flow *s* emitted to environmental compartment *ec* occurring *t* years after rock application in terms of midpoint impact category *m*	unit of midpoint impact[b]/unit of elementary flow[c]
$\boldsymbol{DF_{m,e}^{END}}$	Factor converting midpoint impact *m* to endpoint impact *e*	unit of endpoint impact[d]/unit of midpoint impact[b]
$\boldsymbol{GGR\eta_{t,y}}$	Greenhouse gas removal efficiency *t* years after deployment in year *y*	–

[a] Functional unit: gross geochemical removal of 100 Mt CO_2 in one year.
[b] Unit depends on the midpoint impact category (e.g., kgPM$_{2.5}$ for fine particulate matter formation).
[c] Unit depends on the type of elementary flow (e.g., m^2 for land).
[d] Unit depends on the endpoint impact category (e.g., DALY for human health).

Symbol	Description	Unit
$\boldsymbol{GHG_{s,ec,t,y}}$	Gross amount of greenhouse gas *s* emitted to the atmosphere in year *t* after deployment in year *y*	kg/functional unit[a]
$\boldsymbol{GWP_{s,t}}$	Global warming potential of greenhouse gas *s* emitted in year *t*	$kgCO_2eq/kg$
$\boldsymbol{I_{e,y}^{END}}$	Endpoint impact *e* in year *y*	unit of endpoint impact[d]/functional unit[a]
$\boldsymbol{NR_{s,ec,t,y}}$	Amount of natural resource *s* extracted from environmental compartment *ec* in year *t* after rock application in year *y*	unit of elementary flow[c]/functional unit[a]
$\boldsymbol{P_{s,ec,t,y}}$	Amount of pollutant *s* (excluding greenhouse gases) emitted to environmental compartment *ec* in year *t* after rock application in year *y*	unit of elementary flow[c]/functional unit[a]
$\boldsymbol{PEF_{s,ec,t,y}}$	Amount of elementary flow *s* prevented from being exchanged with environmental compartment *ec* in year *t* after rock application in year *y*	unit of elementary flow[c]/functional unit[a]
$\boldsymbol{Q_{s,ec,t,y}}$	Amount of elementary flow *s* emitted to environmental compartment *ec* in year *t* after rock application in year *y*	unit of elementary flow[c]/functional unit[a]
$\boldsymbol{RF_s}$	Radiative forcing of greenhouse gas *s*	$W/m^2/kg$
$\boldsymbol{t}$	Number of years after deployment	year
$\boldsymbol{t^p}$	Number of years after pulse emission	year
$\boldsymbol{\tau^g}$	Lifetime of CO_2 perturbation in carbon sink *g*	year
$\boldsymbol{\tau_s}$	Lifetime of greenhouse gas *s* in the atmosphere	year
$\boldsymbol{TF_{s,ec}^{C}}$	Toxicity factor quantifying the carcinogenic toxicity effects of metal *s* emitted to environmental compartment *ec*	DALY/kg
$\boldsymbol{TF_{s,ec}^{NC}}$	Toxicity factor quantifying the non-carcinogenic toxicity effects of metal *s* emitted to environmental compartment *ec*	DALY/kg
$\boldsymbol{TH}$	Time horizon	year
$\boldsymbol{TH'}$	Time horizon spanning from the year of the emission to the final year of TH	year

Table S2. Parameters and variables used in the enhanced weathering models.

Symbol	Description	Unit
$\boldsymbol{A}$	Considered time interval	year
$\boldsymbol{C_n^{ALK}}$	Alkalinity concentration in rock *n*	mol/kg
$\boldsymbol{CDR_{d,l,n,t}}$	CO_2 removed during year *t* after a single rock application, dependent on the type of data *d*, location *l* and rock *n*	kg
$\boldsymbol{CDR^{FU}}$	Functional unit: gross amount of CO_2 removed via enhanced weathering reactions within one year	kg/year
$\boldsymbol{Ea_n}$	Activation energy for the weathering of rock *n*	J/mol
$\boldsymbol{P_n}$	Theoretical CO_2 removal potential of rock *n*	$kgCO_2$/kg rock
$\boldsymbol{MM^{CO_2}}$	CO_2 molecular mass	kg/mol
$\boldsymbol{R}$	Universal gas constant	J/mol/K
$\boldsymbol{RM_{d,l,n}^0}$	Mass of rock that must be initially applied to the soil, dependent on the type of data *d*, location *l* and rock *n*	kg
$\boldsymbol{RM_{d,l,n}^A}$	Mass of rock applied annually after the initial year of deployment, dependent on the type of data *d*, location *l* and rock *n*	kg/year
$\boldsymbol{RM_{d,l,n,t}^{CUM}}$	Amount of rock weathered from the time of application until the end of year *t*, dependent on the type of data *d*, location *l* and rock *n*	kg
$\boldsymbol{RM_{d,l,n,t}^{UN}}$	Remaining unweathered rock *t* years after application, dependent on the type of data *d*, location *l* and rock *n*	kg
$\boldsymbol{spy}$	Number of seconds in a year	s/year
$\boldsymbol{SSA_{d,n}}$	Specific surface area, dependent on the type of data *d* and rock *n*	m^2/kg
$\boldsymbol{T^R}$	Reference temperature used for the normalization of rock weathering and alkalinity dissolution rates in this study	K
$\boldsymbol{T_{d,l,n}^0}$	Temperature reported in the literature for rock weathering and alkalinity dissolution rates, dependent on the type of data *d*, location *l* and rock *n*	K
$\boldsymbol{w}$	CO_2 removal efficiency factor (moles of CO_2 removed per mole of alkalinity released)	–
$\boldsymbol{WR_{d,l,n}^{ALK_{T^R}}}$	Alkalinity dissolution rate at 15 °C, dependent on the type of data *d*, location *l* and rock *n*	mol/m^2/s
$\boldsymbol{WR_{d,l,n}^{ALK_{T^0}}}$	Alkalinity dissolution rate reported in the literature at temperature T^0 for type of data *d*, location *l* and rock *n*	mol/m^2/s
$\boldsymbol{WR_{d,l,n}^{ROCK_{T^R}}}$	Rock weathering rate at 15 °C, dependent on the type of data *d*, location *l* and rock *n*	kg/m^2/s
$\boldsymbol{WR_{d,l,n}^{ROCK_{T^0}}}$	Rock weathering rate reported in the literature at temperature T^0, dependent on the type of data *d*, location *l* and rock *n*	kg/m^2/s

2. Methods

Here we describe how we estimated the greenhouse gas removal efficiency and the health and ecosystem impacts.

2.1 Greenhouse gas removal efficiency

The greenhouse gas removal efficiency defined in Equation E1 of the main manuscript is estimated with the time-dependent Global Warming Potential (GWP) of greenhouse gas *s* emitted or removed in year *t*, expressed in kg CO_2eq per kg of gas ($GWP_{s,t}$, Equation SE1).

$GWP_{s,t}$ is derived from the methodology proposed in [1] to estimate dynamic characterization factors for variable time horizons. With this method, the greenhouse gases emitted (or removed) *t* years after the initial deployment will only contribute to (or prevent) global warming during a time horizon TH' contained within the studied 100-year time horizon (TH), estimated as $TH' = TH - t$.

$GWP_{s,t}$ is computed as the ratio between the absolute GWP of greenhouse gas *s* emitted or removed in year *t* and quantified over period TH' ($AGWP_{s,t}^{TH'}$, Equation SE2), and the absolute GWP of CO_2 over a 100-year period ($AGWP_{CO_2}^{TH}$, Equation SE3), both expressed in W·year/m^2/kg. These are calculated with the radiative forcing of the specific greenhouse gas (RF_s, in W/m^2/kg, taken from [2]) and the share of CO_2 (Equation SE4) and other greenhouse gases (Equation SE5) that remains in the atmosphere t^p years after a pulse emission (C_{s,t^p}). The parameters needed to estimate C_{s,t^p} (a_0, a_g, τ_g and τ_s) are taken from [3]

$$GWP_{s,t} = \frac{AGWP_{s,t}^{TH'}}{AGWP_{CO_2}^{TH}} \quad \forall\, s \in J, t \in T \tag{SE1}$$

$$AGWP_{s,t}^{TH'} = \int_{t^p=0}^{t^p=TH-t} RF_s \cdot C_{s,t^p} \cdot dt^p \quad \forall\, s \in J, t \in T \tag{SE2}$$

$$AGWP_{CO_2}^{TH} = \int_{t^p=0}^{t^p=TH} RF_s \cdot C_{s,t^p} \cdot dt^p \quad \forall\, s = CO_2 \tag{SE3}$$

$$C_{s,t^p} = a^0 + \sum_{g=1}^{3} a^g \cdot e^{-\frac{t^p}{\tau^g}} \quad \forall\, s = CO_2, t^p \in T^P \tag{SE4}$$

$$C_{s,t^p} = e^{-\frac{t^p}{\tau_s}} \quad \forall\, s \neq CO_2, t^p \in T^P \tag{SE5}$$

Figure S1 illustrates how the dynamic dimension of the greenhouse gas emissions is accounted for in a hypothetical scenario where 1 tonne (t) carbon dioxide removal (CDR) and 0.5 t CO_2eq occur at $t = 0$, and 0.5 t CO_2 are additionally removed after 25 years. The curves in the figure represent the instantaneous radiative forcing of the CDR and emission pulses, i.e., the global warming impacts (expressed in W·year/m^2) are estimated as the sum of the colored areas between the curves and the x axis, which are negative for CDR and positive for CO_2 emissions. As the figure shows, the prevented impacts of the second CDR pulse are only quantified for the last 75 years of the considered 100-year timeframe and are therefore lower than the prevented

impacts of the pulse emission of the same magnitude occurring at the beginning of the time horizon.

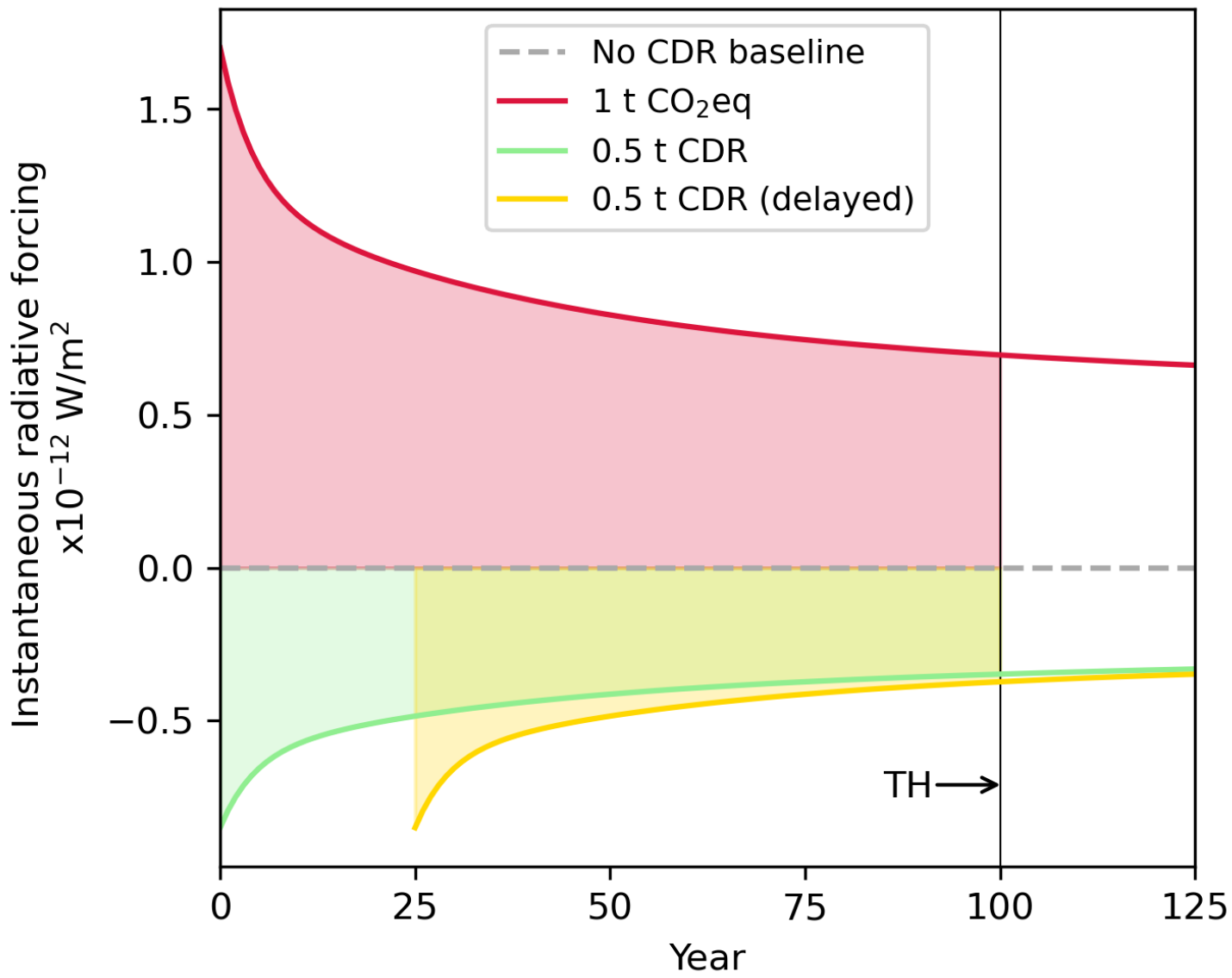

Fig. S1. Instantaneous radiative forcing due to Carbon Dioxide Removal (CDR) and greenhouse gas pulse emissions. The areas under the curves represent the global warming impacts (in W·year/m^2) over a 100-year time horizon (TH).

2.2 Health and ecosystem impacts

We estimated the impacts of our scenarios on human health and ecosystems with the ReCiPe 2016[4] impact assessment method, which establishes a linear relationship between the damage to these endpoint areas of protection and the elementary flows (natural resources and emissions) in the life cycle inventories.

We calculated the impacts at the midpoint level–those related to environmental mechanisms such as particulate matter formation or land use–by multiplying the amount of elementary flow *s* exchanged with the environment due to the rock application in year *y* ($Q_{s,ec,t,y}$) by the elementary flow's characterization factor for midpoint impact category *m* ($CF^{MID}_{m,s,ec,t}$). Notably, when the health and ecosystem damage linked to the global warming midpoint impact category are quantified, the characterization factor $CF^{MID}_{m,s,ec,t}$ equals the $GWP_{s,t}$ defined in Equation SE1.

Consistent with our goal of quantifying the impacts of CDR in year *y* (2030 or 2050), we only consider the elementary flows happening during that year, i.e., the initial year of the considered time horizon ($t = 0$). Since CDR happens progressively as rock particles weather, emissions occurring after the initial year of rock application are allocated to subsequent CDR.

We applied a factor $DF^{END}_{m,e}$ to transform midpoint impacts into impacts on the endpoint area of protection *e* (damage to human health or ecosystems). As Equation SE6 indicates, we calculate the endpoint impacts of the CDR occurring in year *y*, $I^{END}_{e,y}$, as the sum of the endpoint impacts quantified over the selected time horizon.

$$I_{e,y}^{END} = \sum_{s\in S} \sum_{ec\in EC} \sum_{m\in M} Q_{s,ec,t,y} \cdot CF_{m,s,ec,t}^{MID} \cdot DF_{m,e}^{END} \quad \forall\, t = 0, e \in E, y \in Y \tag{SE6}$$

The elementary flows $Q_{s,ec,t,y}$ (defined in Equation SE7) are those exchanged with the environment throughout the rock supply chains, and include the removed CO_2 ($CDR_{s,ec=air,t,y}$), greenhouse gas emissions ($GHG_{s,ec=air,t,y}$), other pollutants ($P_{s,ec,t,y}$) and natural resources ($NR_{s,ec,t,y}$), plus the elementary flows prevented from being emitted or extracted as a result of the fertilization effects associated with the released phosphorus. Note that the values of the elementary flows removed or prevented ($CDR_{s,ec=air,t,y}$ and $PEF_{s,ec,t,y}$) are ≤0.

$$Q_{s,ec,t,y} = CDR_{s,ec=air,t,y} + GHG_{s,ec=air,t,y} + P_{s,ec,t,y} + NR_{s,ec,t,y} + PEF_{s,ec,t,y} \quad \forall\, s \in S,\ ec \in EC, t \in T, y \in Y \tag{SE7}$$

The ReCiPe 2016 impact assessment method handles uncertainty by defining three cultural perspectives that group different sources of uncertainty and value choices. While the main manuscript focuses primarily on the hierarchist middle-ground perspective, toxicity impacts calculated for alternative perspectives are provided in the Supporting results section. Cultural perspectives are defined as follows:

- Individualist (20-year time horizon). It quantifies short-term impacts based on a very high level of evidence and technological optimism regarding human adaptation. Humans are only exposed to metals through drinking water and air.
- Hierarchist (100-year time horizon). The considered timeframe and plausibility of impact mechanisms are based on the scientific consensus. It considers all exposure routes.
- Egalitarian (infinite time horizon). It reflects the most precautionary approach for damage modeling, considering all exposure routes.

The metal toxicity factors $TF_{s,ec}^{C}$ and $TF_{s,ec}^{NC}$ in Equation E2 of the main manuscript are calculated as the product of $CF_{m,s,ec,t}^{MID}$ and $DF_{m,e}^{END}$ for the human health (*H*) endpoint and the carcinogenic (*C*) and non-carcinogenic (*NC*) toxicity midpoint impact categories, respectively (Equations SE8 and SE9).

$$TF_{s,ec}^{C} = CF_{m,s,ec,t}^{MID} \cdot DF_{m,e}^{END} \quad \forall\, s \in ME, ec \in EC, m = C, e = H, t = 0 \tag{SE8}$$
$$TF_{s,ec}^{NC} = CF_{m,s,ec,t}^{MID} \cdot DF_{m,e}^{END} \quad \forall\, s \in ME, ec \in EC, m = NC, e = H, t = 0 \tag{SE9}$$

The characterization factors provided by the ReCiPe 2016 method to quantify human toxicity and ecotoxicity are derived from the USES-LCA 2.0[5] model and the USETOX[6] database. These global toxicity factors are directly proportional to the product of fate, exposure, and effect factors, described below.

- Fate factors represent the environmental persistence of a substance, quantified as the marginal change in the dissolved steady state concentration in an environmental compartment due to a marginal emission change[7].
- Exposure factors account for the fraction of a substance in a given environmental compartment that is bioavailable. Human exposure occurs through inhalation or oral uptake (via food or drinking water) and varies across geographical scales. Regional exposure factors are ultimately aggregated into a global, population-weighted average. Notably, dietary ingestion dominates human metal intake in the long term; it accounts for 98.7% and 86.7%

(median) of the intake fractions when metals are emitted to agricultural and natural soils, respectively. For metal emissions to seawater, exposure through food represents 100% of the intake fraction[7]. Specifically, metals accumulate in food products through three mechanisms: 1) crops take up metals from soil, 2) livestock ingest metals in feed, and 3) fish absorb metals from contaminated waters[7]. For ecosystems, exposure factors represent the dissolved fraction of a substance, which is assumed to be bioavailable[6].

– Effect factors quantify the toxicity effects of the emitted substance, derived from human and ecological toxicity data[8].

3. Enhanced weathering models

Next, we summarize the key equations and variables, data sources, and main assumptions and limitations of our models.

3.1 Equations and variables

The enhanced weathering life cycle inventories are estimated with two sets of data (d): experimental (exp) and theoretical (th), differentiating between the rock type *n* (basalt or dunite) and the location *l* of the rock application (inland or coastal).

We used the Arrhenius equation to calculate the alkalinity dissolution and rock weathering rates at a reference temperature T^R of 15 °C ($WR_{d,l,n}^{ALK_{T^R}}$ and $WR_{d,l,n}^{ROCK_{T^R}}$, expressed in mol/m^2/s and kg/m^2/s, respectively) based on the alkalinity dissolution and rock weathering rates ($WR_{d,l,n}^{ALK_{T^0}}$ and $WR_{d,l,n}^{ROCK_{T^0}}$) reported in the literature for temperature $T_{d,l,n}^0$, and considering activation energy (Ea_n) values of 5·10^4 J/mol for basalt[9] and 6.09·10^4 J/mol for dunite[10] (Equations SE10 and SE11). The temperature-normalized results are compiled in Tables 1.3 and 1.4 of the Supporting Data[11].

$$WR_{d,l,n}^{ALK_{T^R}} = WR_{d,l,n}^{ALK_{T^0}} \cdot e^{-\frac{Ea_n}{R}\cdot\left(\frac{1}{T^R}-\frac{1}{T_{d,l,n}^0}\right)} \quad \forall\, d \in exp, l \in L, n \in N \qquad \text{(SE10)}$$

$$WR_{d,l,n}^{ROCK_{T^R}} = WR_{d,l,n}^{ROCK_{T^0}} \cdot e^{-\frac{Ea_n}{R}\cdot\left(\frac{1}{T^R}-\frac{1}{T_{d,l,n}^0}\right)} \quad \forall\, d \in th, l \in L, n \in N \qquad \text{(SE11)}$$

To calculate the mass of rock that must be initially applied to the soil ($RM_{d,l,n}^0$) in the inland and coastal enhanced weathering scenarios based on experimental data, we used Equation SE12, where CDR^{FU} represents the functional unit (i.e., the amount of CO_2 removed within one year, expressed in kg/year), $SSA_{d,n}$ is the rock specific surface area (SSA, m^2/kg), spy is the number of seconds in a year, and MM^{CO_2} is the CO_2 molecular mass (kg/mol).

Previous studies have found that the dissolution of the silicate minerals is the rate limiting step of the weathering reactions[12]; hence, we assumed that the dissolved minerals can immediately react with the dissolved CO_2. We adjusted the stoichiometric CDR (one mole of atmospheric CO_2 reacts with one mole of alkalinity released from the rock) with a CDR efficiency factor (w) of 0.775 (typically between 0.7 and 0.85)[13] to account for the conversion of some bicarbonate anions to carbonate anions, which occurs under conditions of excess alkalinity and releases part of the sequestered CO_2. While prior studies estimate the fraction of rock dissolved with the shrinking core model[12,14], it has been criticized for considering perfect spherical particles rather than highly irregular grains, thereby underestimating the SSA and leading to longer dissolution times[15,16]. Hence, we opt for considering a constant SSA, consistent with previous analyses[15,17].

In the scenarios based on theoretical data, we estimated $RM_{d,l,n}^0$ with Equation SE13, dividing the annual CDR target by the product of the rock fraction that is weathered in one year and the theoretical rock CDR potential P_n (kgCO_2/kg rock), which is directly proportional to the rock's alkalinity content and accounts for the CDR efficiency factor.

$$RM^{0}_{d,l,n} = \frac{|CDR^{FU}|}{WR^{ALK_{T}R}_{d,l,n} \cdot SSA_{d,n} \cdot spy \cdot w \cdot MM^{CO_2}} \; \forall\, d = exp, l \in L, n \in N \qquad \text{(SE12)}$$

$$RM^{0}_{d,l,n} = \frac{|CDR^{FU}|}{WR^{ROCK_{T}R}_{d,l,n} \cdot SSA_{d,n} \cdot spy \cdot P_n} \; \forall\, d = th, l \in L, n \in N \qquad \text{(SE13)}$$

We use Equation SE14 to estimate the amount of rock that must be applied every year after the initial deployment ($RM^{A}_{d,l,n}$) to maintain a constant annual CDR rate. Since the amount of alkalinity dissolved is directly proportional to the SSA, maintaining a constant CDR rate requires keeping the total rock surface area constant over time. Hence, we assume that the annual rock application equals the mass of rock that weathered during the previous year, estimated with $WR^{ROCK_{T}R}_{d,l,n}$. For scenarios based on experimental data, $WR^{ROCK_T}_{d,l,n}$ is calculated with Equation SE15 based on the alkalinity concentration in the rock, C^{ALK}_{n} (mol/kg).

$$RM^{A}_{d,l,n} = RM^{0}_{d,l,n} \cdot WR^{ROCK_{T}R}_{d,l,n} \cdot SSA_{d,n} \cdot spy \; \forall\, d \in D, l \in L, n \in N \qquad \text{(SE14)}$$

$$WR^{ROCK_{T}R}_{d,l,n} = \frac{WR^{ALK_{T}R}_{d,l,n}}{C^{ALK}_{n}} \; \forall\, d = exp, l \in L, n \in N \qquad \text{(SE15)}$$

To calculate the dynamic CDR profile resulting from a single rock deployment, we use Equations SE16-SE21. These equations describe how as the initially applied rock gradually weathers, the remaining rock available for weathering decreases, thereby lowering the annual CDR.

The amount of CO_2 removed during year *t* after the rock application ($CDR_{d,l,n,t}$, expressed in kg) is initially 0 (Equation SE16). In subsequent years, $CDR_{d,l,n,t}$ is directly proportional to the mass of rock that weathers in that specific year, as estimated by Equations SE17 and SE18 for scenarios based on experimental and theoretical datasets, respectively.

Calculating the latter requires previously computing the cumulative amount of rock weathered from the time of application until the end of year *t* ($RM^{CUM}_{d,l,n,t}$, in kg). This value is set to 0 initially (Equation SE19) and calculated for subsequent years using Equation SE20, which considers the weathering rates reported in the literature and the remaining amount of rock unweathered at the end of the preceding year. The mass of rock that remains unweathered in year *t* after the rock application ($RM^{UN}_{d,l,n,t}$, in kg) is calculated with Equation SE21.

$$CDR_{d,l,n,t} = 0 \;\; \forall\, d \in D, l \in L, n \in N, t = 0 \qquad \text{(SE16)}$$

$$CDR_{d,l,n,t} = RM^{UN}_{d,l,n,t-1} \cdot WR^{ALK_{T}R}_{d,l,n} \cdot SSA \cdot spy \cdot A \cdot w \cdot MM^{CO_2} \qquad \text{(SE17)}$$
$$\forall\, d = exp, l \in L, n \in N, t > 0$$

$$CDR_{d,l,n,t} = P_n \cdot (RM^{CUM}_{d,l,n,t} - RM^{CUM}_{d,l,n,t-1}) \;\; \forall\, d = th, l \in L, n \in N, t > 0 \qquad \text{(SE18)}$$

$$RM^{CUM}_{d,l,n,t} = 0 \;\; \forall\, d \in D, l \in L, n \in N, t = 0 \qquad \text{(SE19)}$$

$$RM^{CUM}_{d,l,n,t} = RM^{UN}_{d,l,n,t-1} \cdot WR^{ROCK_{T}R}_{d,l,n} \cdot SSA \cdot spy \cdot A + RM^{CUM}_{d,l,n,t-1} \qquad \text{(SE20)}$$
$$\forall\, d \in D, l \in L, n \in N, t > 0$$

$$RM^{UN}_{d,l,n,t} = RM^{0}_{d,l,n} - RM^{CUM}_{d,l,n,t} \; \forall\, d \in D, l \in L, n \in N, t \in T \qquad \text{(SE21)}$$

3.2 Scenario parameters

Table S3 summarizes the key characteristics of the studies upon which the enhanced weathering scenarios were modeled. The parameters derived from these works are described in further detail below.

We modeled two coastal enhanced weathering scenarios based on the total alkalinity dissolution rates derived from laboratory experiments[18,19] where olivine particles were added to the seawater and subjected to continuous rotation movements to simulate the weathering conditions in coastal environments. In [18], experiments were conducted at 17 °C, and olivine particles had an SSA of 0.295 m^2/g and a median size (p_{50}) of 143 µm, with the size below which 80% of particles fall (p_{80}) ≈200 µm. We calculated the amount of dunite applied considering that olivine makes up 90%[20] of the rock, and an alkalinity content of 20.02 mol/kg dunite[18]. In the coastal enhanced weathering scenarios based on experimental data from [18], we assume that all the nickel contained in the applied dunite is released in the year following rock application, consistent with the high nickel dissolution rates reported.

In the scenarios modeled after the experiments described in [19], which were conducted at 15 °C, p_{50} is 136±1 µm, p_{80} equals 186±2 µm, and the SSA is 0.9±0.1 m^2/g. The olivine share in dunite was 90.54%, and the alkalinity content was 23.31 mol/kg dunite. We also considered the nickel dissolution rates provided in both studies[18,19], and assessed how uncertainties in alkalinity and nickel dissolution rates[18,19] (compiled in Tables 1.3 and 1.5 in the Supporting Data[11]) affected our results. In both coastal enhanced weathering scenarios, we considered an application rate of 22 kg/m^2, as in [20]. This corresponds to the largest application rate across the experimental studies simulating inland conditions. It was selected under the assumption that coastal scenarios permit higher application rates because wave action and tidal transport accelerate the movement of rock particles into the marine environment.

The inland scenarios deploying dunite are based on a mesocosm[20] study conducted at ≈15 °C and a laboratory[21] experiment at 19 °C. Weathering rates were estimated from the amount of magnesium initially contained in the rock that is dissolved during the experiments. In the scenario modeled after [20], we derive data from the experimental setup based on fine dunite grains with a 90% olivine content and a magnesium-related alkalinity concentration of 22.04 mol per kg dunite, considering an application rate of 22 kg/m^2 to the soil. The rock particle data are as follows. Dominant particle size class: 25.5 µm, p_{80}: 43.5 µm, SSA: 12.1 m^2/g (range: 11.8–12.5 m^2/g). For the uncertainty analysis, we estimate the range of alkalinity dissolution rates based on the reported alkalinity concentrations at the outlet of the experimental setup.

In the inland scenario based on data from [21], the application rate is 12.9 kg/m^2. The dominant particle size class is 125-212 µm for dunite particles with an SSA of 3.04±0.03 m^2/g, and we estimate a p_{80} of 368 µm. We assume that 90% of the dunite content is olivine, consistent with [20]. The alkalinity content associated with magnesium is 21.31 mol per kg dunite. Our uncertainty analysis considers the reported range of alkalinity dissolution rates. We assume 0.1 g P_2O_5 per kg of rock across all the dunite scenarios[20,21].

The experimental basalt scenarios are based on data from [22] and [23], both considering a rock application rate of 5 kg/m^2. The alkalinity dissolution rates of the basalt scenarios are compiled

in Table 1.3 of the Supporting Data[11]. In the scenarios modeled after data from soil core experiments[22] at an average temperature of 12 °C, the mass of basalt needed is computed with the alkalinity dissolution rates–estimated from the calcium, magnesium and sodium released in the experiments–and the rock alkalinity content (10.4 mol/kg). Here, the dominant particle size class is 63-250 µm (p_{80}≈ 250 µm), the SSA is 9.23 ± 0.076 m^2/g, and the P_2O_5 concentration is 9 g P_2O_5 per kg of basalt. We analyzed the influence of the reported uncertainty in the alkalinity release rate on our results.

The basalt particles in the scenario considering results from a field trial[23] present a grain size p_{80} of 267 µm and an SSA of 1.02±0.014 m^2/g[24]. Since this study does not provide alkalinity dissolution rates, we derived them from the reported CDR over a four-year period at an average temperature of 10.9 °C[25]. We used a value of $5.25\cdot10^{-2}$ kg CO_2 per kg basalt to solve Equation SE12 for $WR_{d,l,n}^{ALK_T}$ (range considered for the uncertainty analysis: $3.35\cdot10^{-2}$-$7.15\cdot10^{-2}$ kg CO_2 per kg basalt). The alkalinity (calcium and magnesium) content in the applied rock is 7.37 mol per kg of basalt, whereas the P_2O_5 concentration is 1.98 g P_2O_5 per kg of basalt[24].

In the theoretical scenarios, we took the rock weathering rates (and their corresponding uncertainty ranges) estimated in [15] (Table 1.4 in the Supporting Data[11]) . We considered the application of 15 kg/m^2 of rock particles with a uniform size of 25 µm and estimated the SSA (1.28 m^2/g) with the power function provided by [15]. We selected this particle size because it corresponds to the lower end of the dominant class sizes across the experimental studies. Since smaller particles present higher SSAs, leading to higher CDR rates, the theoretical scenarios serve as a high-performance benchmark against which the experimental scenarios can be measured. The assumed CDR potential P_n of dunite and basalt is 0.95 and 0.3 kg CO_2 per kg of rock, respectively[15], while the considered P_2O_5 concentration in the theoretical scenarios is 0.1 g per kg of dunite [20,21] and 2.36 g per kg of basalt[26].

We calculated the electricity demand for the rock crushing and grinding operations using the correlation between the particle size and the energy consumption provided in [15]. We assumed an average road transportation distance of 215 km (range: 180-250 km)[27], based on scenarios linking rock extraction sites and croplands in the United Kingdom. Finally, our inventories do not include land occupation or transformation elementary flows in the foreground system because croplands, forestlands and coastal areas continue providing the same services as prior to the rock deployment.

Table S3. Main characteristics of the source studies used for the enhanced weathering scenarios. Data gaps (in *italics*) were taken from other studies or assumed *(a)* by the authors.

	**Experimental data**						**Theoretical data**
	**Dunite (D)**				**Basalt (B)**		**D/B**
	Coastal		Inland		Inland		
	Laboratory	Laboratory	Mesocosm	Laboratory	Mesocosm	Field	–
	Montserrat et al. (2017)[18]	Flipkens et al. (2023)[19]	Amann et al. (2020)[20]	Renforth et al. (2015)[21]	Vienne et al. (2022)[22]	Beerling et al. (2024)[23]	Strefler et al. (2018)[15]
Temperature T^0 (°C)	17	15	15	19	12	10.9[25]	25
p_{80} (µm)	200	186	43.5	368	250	267	*25 (a)*
p_{50} (µm)	143	136	–	–	–	–	*25 (a)*
Dominant class (µm)	–	–	25.5	125-212	63-250	–	*25 (a)*
SSA (m^2/g)	0.295	0.9	12.1	3.04	9.23	1.02	1.28
Alkalinity (mol/kg rock)	20.02	23.21	22.04	21.31	10.4	7.37	25.40 (D), 8.02 (B)
P_2O_5 (g/kg rock)	*0.1*[20,21]	*0.1*[20,21]	0.1	0.1	9	1.98	*0.1*[20,21] (D), 2.36[26] (B)
Application rate (kg/m^2)	*22*[20]	*22*[20]	22	12.9	5	5	15
Initial pH	7.9-8.2	8.2	6.6	7.5[e]	7.72	6.1	7
Soil properties	–	–	Sandy laom	Calcareous	Loamy	Silt loam	–

[e] pH of the control effluent solution used as proxy.

3.3 Main assumptions and limitations

The functional unit–defined as the gross removal of 100 Mt CO_2 in one year–accounts for 26% and 6% of the 2030 and 2050 CDR targets, respectively, within the selected climate change mitigation scenario (SSP2-PkBudg1000, projected with the REMIND 3.5 integrated assessment model[28,29]).

While this value represents a conservative fraction of the theoretical global potential of enhanced weathering (0.35-2.00 Gt CO_2 per year by 2050, according to previous techno-economic analyses[30,31]), other CDR methods are more likely to scale up faster since they are currently better understood and present higher maturity levels[32]. Hence, the functional unit represents an ambitious yet realistic benchmark.

Ideally, the performance of enhanced weathering trials deploying dunite and basalt should be assessed under equal experimental conditions to enable a fair comparison. This was not possible due to the limited body of experimental results reported in the literature. However, the scenarios based on experimental data allowed us to illustrate the performance gap between the theoretical and experimental weathering rates (discussed in the main text).

To estimate the metal emissions to the soil (inland scenarios) and seawater (coastal scenarios), we considered the metal concentrations in basalt reported in [33–35], and took those of dunite from [20] (Tables 1.1 and 1.2 in the Supporting Data[11]). Although some experiments simulating inland enhanced weathering conditions found slower dissolution rates for certain elements relative to the alkaline metals driving CDR[20,21,36], they did not quantify the accumulation of metals in the soil surface, which could be substantial[37]. Furthermore, metals can potentially be released due to changes in the soil pH or redox conditions[38,39], hard to control in an open field. Thus, our uncertainty analysis considered that metals are released from the weathered rocks at rates ranging between 0% (total retention) and 100% (complete mobilization), allocating the impacts of the elementary flows occurring on a given year to that year's CDR.

Moreover, while we consider global toxicity factors, metal bioavailability is highly dependent on the soil conditions[40], and therefore the application of rock particles to specific locations could lead to varying toxicity effects based on site-specific parameters. Toxicity outcomes are also sensitive to assumptions regarding human adaptation; for instance, factoring in the deployment of advanced water potabilization technologies highly efficient at metal removal could decrease the human intake fractions via drinking water.

Our models do not account for the potential emission of fine particulate matter that could occur during grinding processes[16]. However, because the dominant particle size class considered in our scenarios is significantly larger (≥25 µm) than the hazardous threshold considered by the ReCiPe 2016 method (≤2.5 µm), we expect the potential contribution of fine particulates to the total health effects of our scenarios to be minimal.

On the other hand, we consider that the release of phosphorus contributes to biomass fertilization. However, our model does not account for the potential implications of concurrently applying nitrogen fertilizers and rock particles to the soil; while enhanced weathering could partially abate fertilizer-derived N_2O emissions, chemical reactions could be driven by HNO_3 instead of the dissolved CO_2. Consequently, the presence of this and other soil acidic species

could undermine CDR efficiency[41,42], a phenomenon excluded from our models due to data limitations.

Furthermore, enhanced weathering could lead to additional CDR–not quantified here due to data limitations–through an increase in soil organic carbon[43,44], or improved soil structure and water retention[45]. Another limitation of our models is that they do not reflect the potential co-benefits of counteracting soil and ocean acidification[46], as the ReCiPe 2016[4] impact assessment method lacks characterization factors to quantify the alkalinity-induced neutralization effects of enhanced weathering.

Finally, our inventories are based on global market activities, which implies that the deployment of enhanced weathering in specific locations could lead to different results due to differences in the inventory activities such as the electricity mixes.

4. Supporting results

Here we present additional results to complement the discussion in the main manuscript. Figure S2 shows the time-dependent global warming impacts of our scenarios expressed per t of rock deployed. Figure S3 displays the fraction of rock particles weathered over time following the initial application.

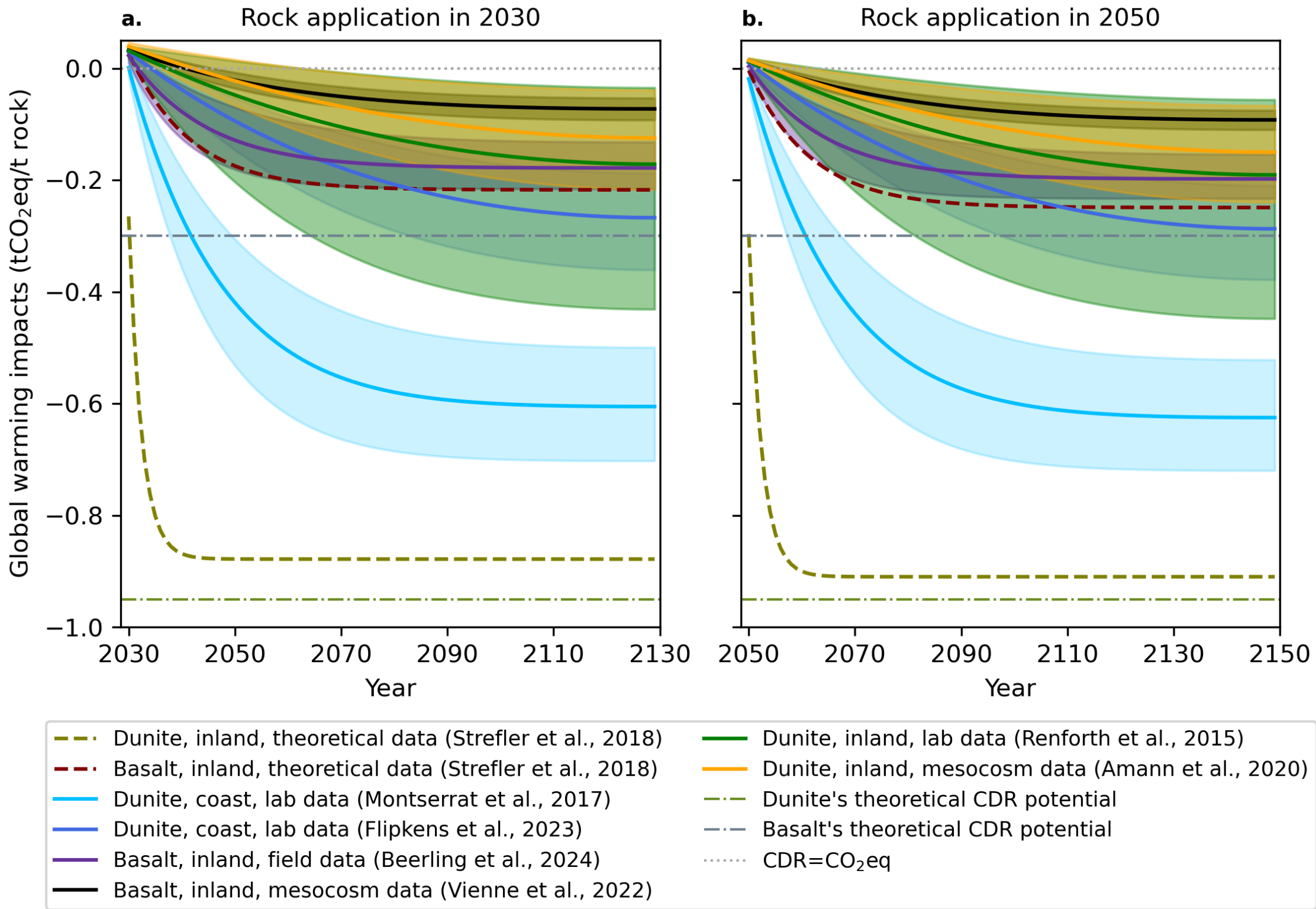

Fig. S2. Evolution of global warming impacts associated with the deployment of 1000 kg of rock at the beginning of the 100-year time horizon, considering experimental[18–23] and theoretical[15] data for the application of dunite and basalt on coastal and inland environments. The shaded areas represent the uncertainty in the results. **a** Single rock application in 2030. **b** Single rock application in 2050.

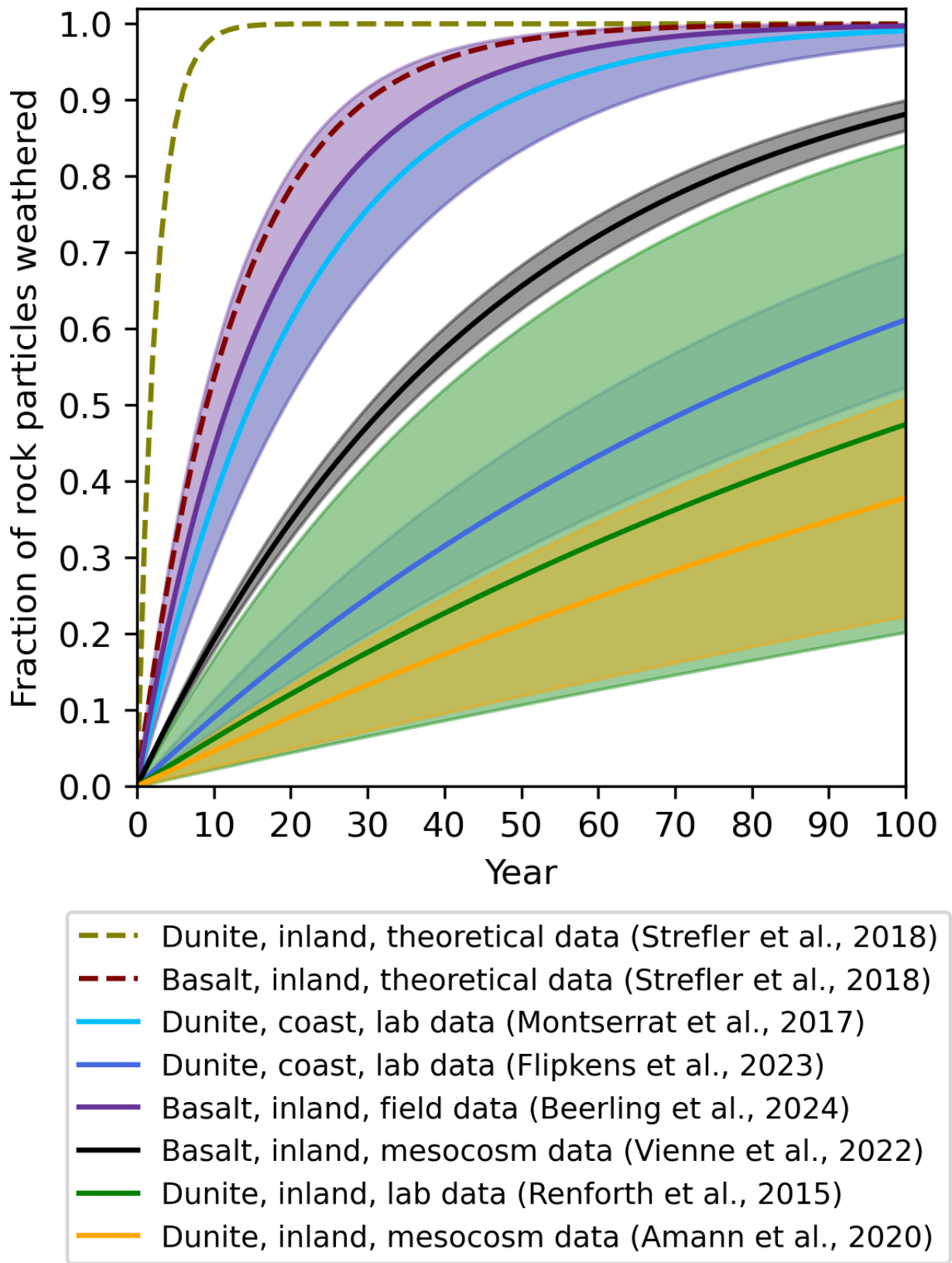

Fig. S3. Fraction of rock particles weathered over 100 years following a single initial application, considering experimental[18–23] and theoretical[15] data for the application of dunite and basalt on coastal and inland environments. The shaded areas represent the uncertainty in the results.

Table S4 shows the number of years required to achieve complete weathering in each scenario.

Table S4. Number of years required to achieve complete weathering in each scenario.

**Scenario**	**Years to achieve complete weathering**
Dunite, inland, theoretical data (Strefler et al., 2018)[15]	14
Basalt, inland, theoretical data (Strefler et al., 2018)[15]	70
Dunite, coast, lab data (Montserrat et al., 2017)[18]	113 (91, 147)
Dunite, coast, lab data (Flipkens et al., 2023)[19]	561 (442, 716)
Basalt, inland, field data (Beerling et al., 2024)[23]	91 (64, 148)
Basalt, inland, mesocosm data (Vienne et al., 2022)[22]	249 (231, 270)
Dunite, inland, lab data (Renforth et al., 2015)[21]	824 (288, 2350)
Dunite, inland, mesocosm data (Amann et al., 2020)[20]	1115 (746, 2099)

Figures S4 and S5 illustrate the greenhouse gas removal efficiencies and endpoint (human health and ecosystem) impacts, respectively, for enhanced weathering scenarios aligned with alternative climate change mitigation pathways.

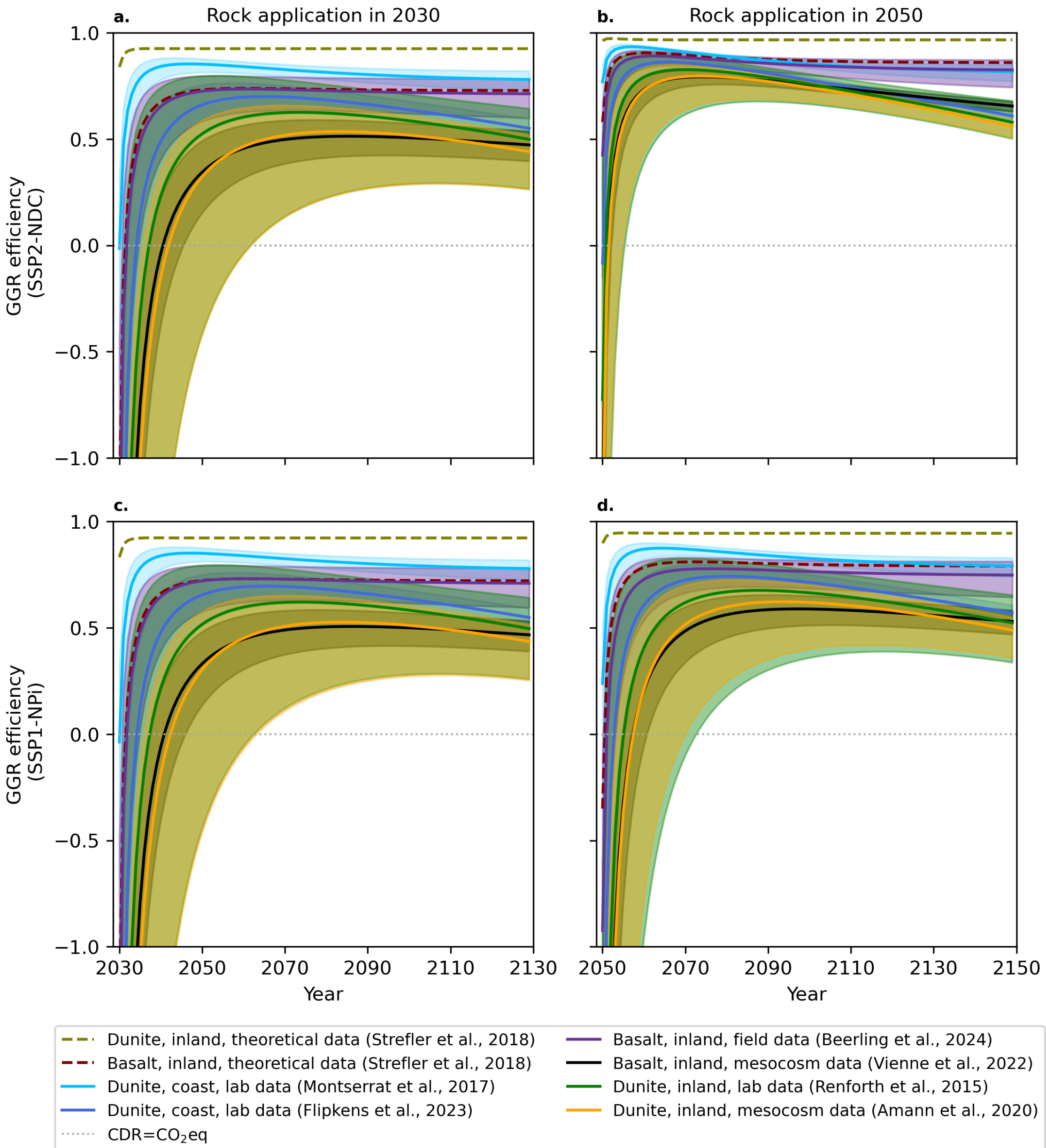

Fig. S4. Evolution of greenhouse gas removal (GGR) efficiencies over 100 years, considering experimental[18–23] and theoretical[15] data for the application of dunite and basalt on coastal and inland environments. The shaded areas represent the uncertainty in the results. **a** Single rock application in 2030, inventory data aligned with SSP2-NDC scenario. **b** Single rock application in 2050, inventory data aligned with SSP2-NDC scenario. **c** Single rock application in 2030, inventory data aligned with SSP1-NPi scenario. **d** Single rock application in 2050, inventory data aligned with SSP1-NPi scenario.

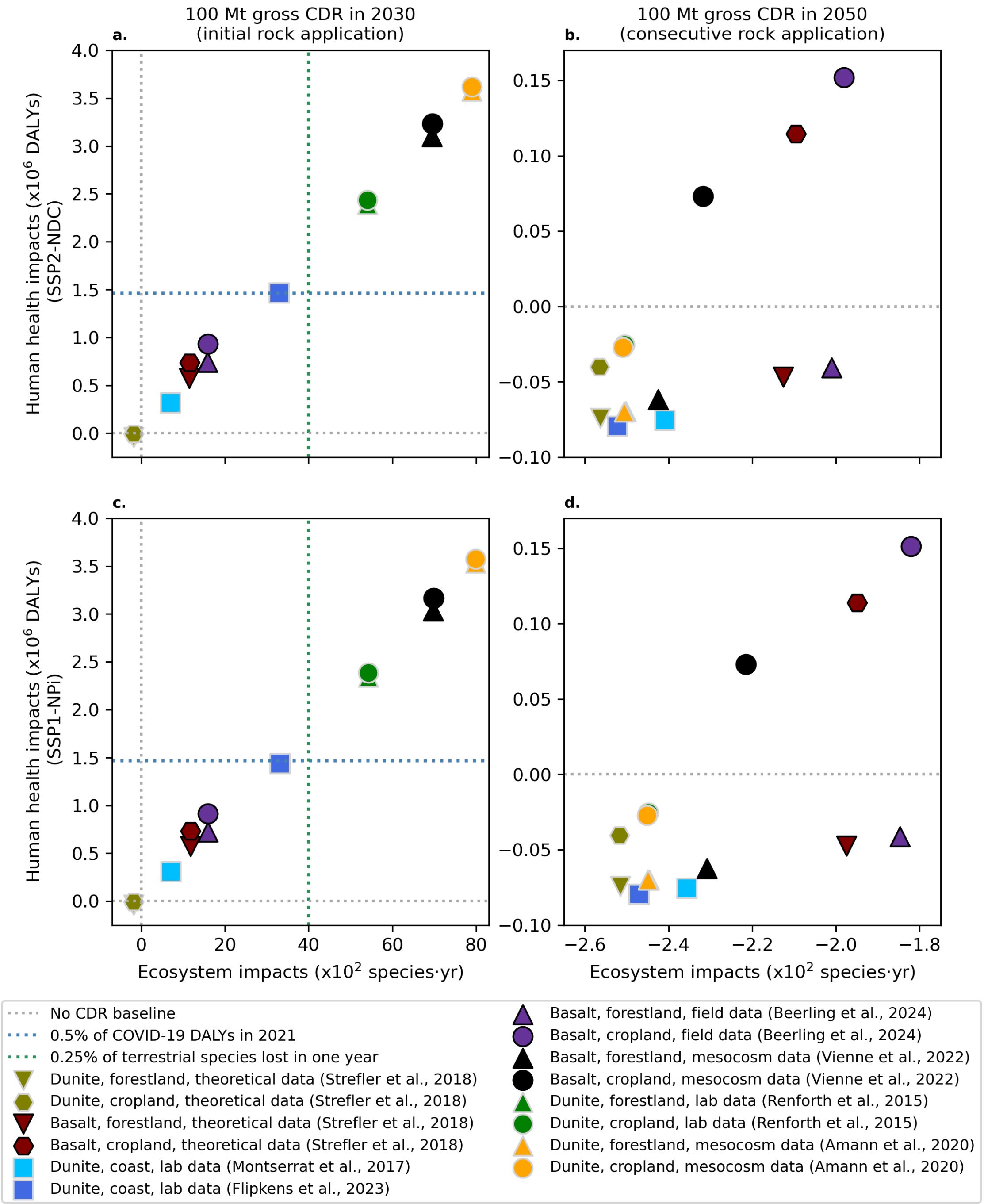

Fig. S5. Human health and ecosystem impacts of removing 100 Mt CO_2 (gross) in 2030 and 2050, considering experimental[18–23] and theoretical[15] data for the application of dunite and basalt on coastal and inland (forestlands and croplands) environments**. a** Rock application in 2030 for the first time, inventory data aligned with SSP2-NDC scenario. **b** Consecutive rock application in 2050, inventory data aligned with SSP2-NDC scenario. **c** Rock application in 2030, inventory data aligned with SSP1-NPi scenario. **d** Consecutive rock application in 2050, inventory data aligned with SSP1-NPi scenario.

Figure S6 depicts the breakdown of health and ecosystem impacts for a coastal enhanced weathering scenario, while Figure S7 shows the disaggregated ecosystem impacts of two inland enhanced weathering scenarios.

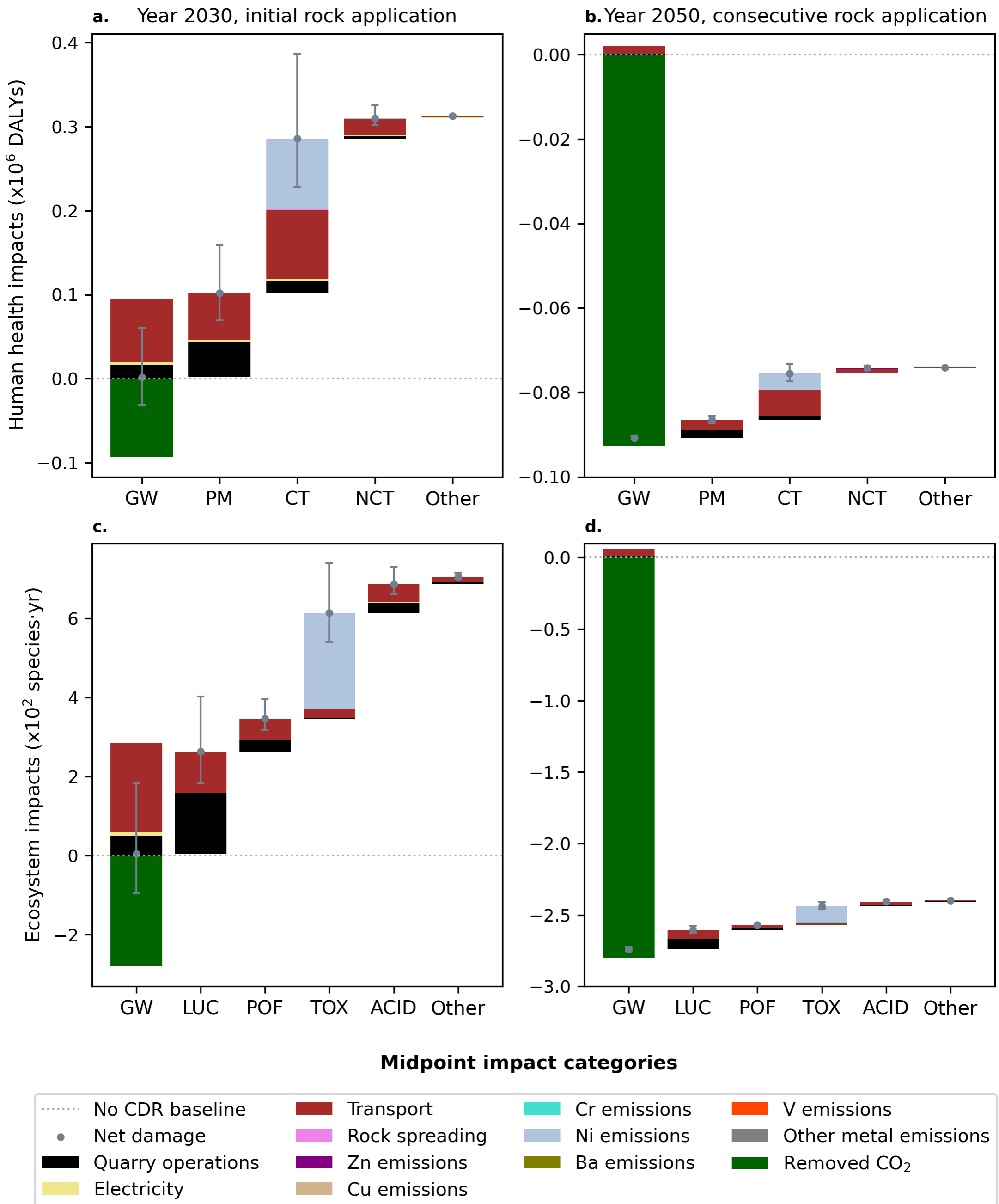

Fig. S6. Health and ecosystem impacts of removing 100 Mt CO_2 (gross) in 2030 and 2050 through coastal enhanced weathering, considering initial rock application in 2030 and consecutive applications in subsequent years. Data from [18]. Error bars represent the uncertainty in the results. Impacts disaggregated by the contribution of i) unit processes and direct emissions, and ii) environmental mechanism leading to health–global warming (GW), fine particulate matter formation (PM), carcinogenic toxicity (CT), non-carcinogenic toxicity (NCT), and others (tropospheric ozone formation, stratospheric ozone depletion, ionizing radiation, and water consumption)–and ecosystem impacts–GW, land-use change (LUC), photochemical ozone formation (POF), ecotoxicity (TOX), terrestrial acidification (ACID) and others (water consumption and eutrophication). **a** Health impacts of dunite application, 2030. **b** Health impacts of dunite application, 2050. **c** Ecosystem impacts of dunite application, 2030. **d** Ecosystem impacts of dunite application, 2050.

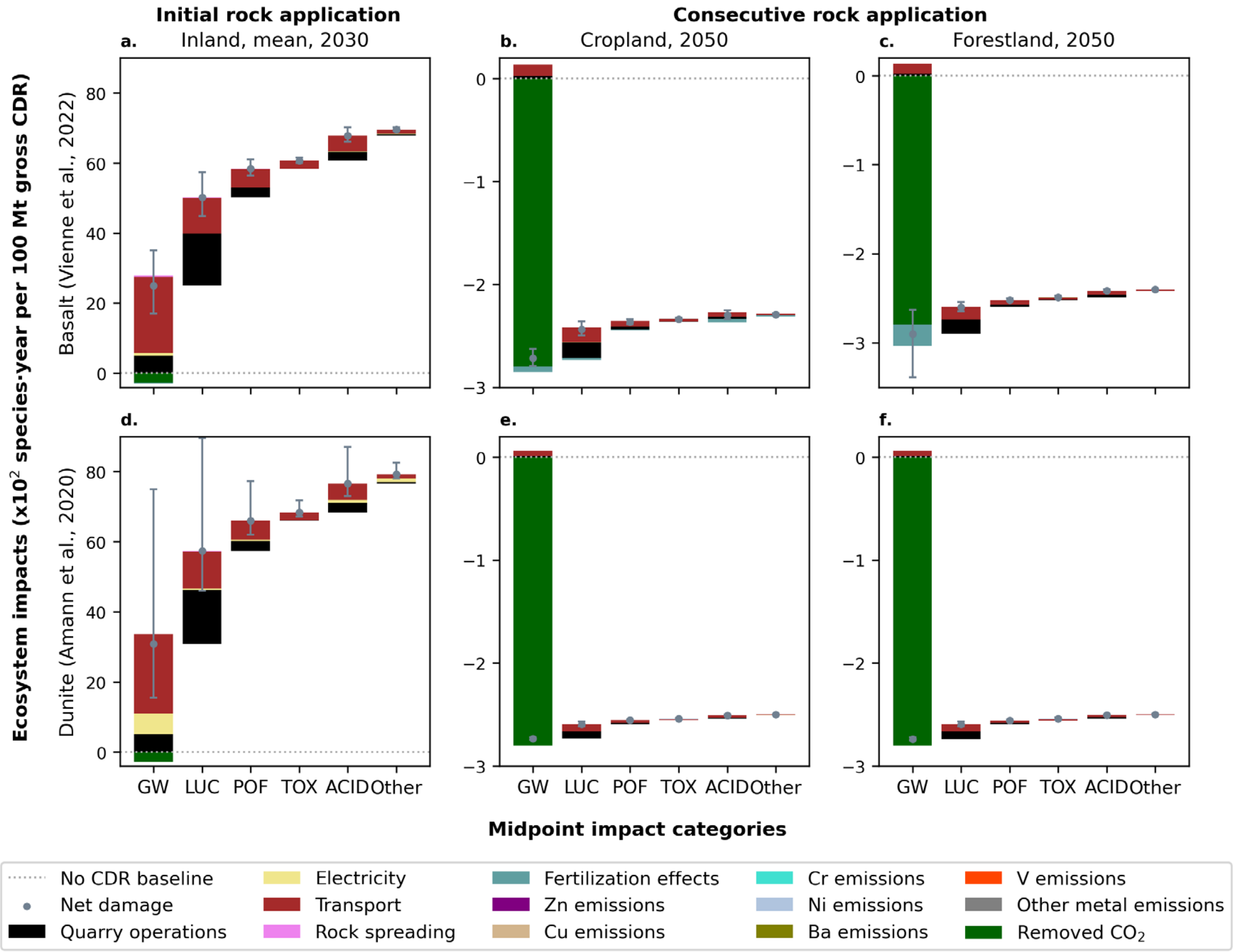

Fig. S7. Ecosystem impacts of removing 100 Mt CO_2 (gross) in 2030 and 2050, considering initial rock application in 2030 and consecutive applications in subsequent years. Data from [20,22]. Error bars represent the uncertainty in the results. Impacts disaggregated by the contribution of i) unit processes and direct emissions, and ii) environmental mechanisms: global warming (GW), land-use change (LUC), photochemical ozone formation (POF), ecotoxicity (TOX), terrestrial acidification (ACID) and others (water consumption and eutrophication). **a** Basalt application to cropland/forestland (mean), 2030. **b** Basalt application to cropland, 2050. **c** Basalt application to forestland, 2050. **d** Dunite application to cropland/forestland (mean), 2030. **e** Dunite application to cropland, 2050. **f** Dunite application to forestland, 2050.

Figure S8 shows the health and ecosystem impacts of our scenarios considering an initial application of 1 Mt of rock particles in 2030 and consecutive annual rock applications to maintain the scenario-specific CDR rate constant, while Figure S9 displays the disaggregated health impacts of two representative scenarios relative to the same functional unit. Figure S10 displays the rock toxicity potentials, expressed in DALYs per Mt of rock weathered within one year.

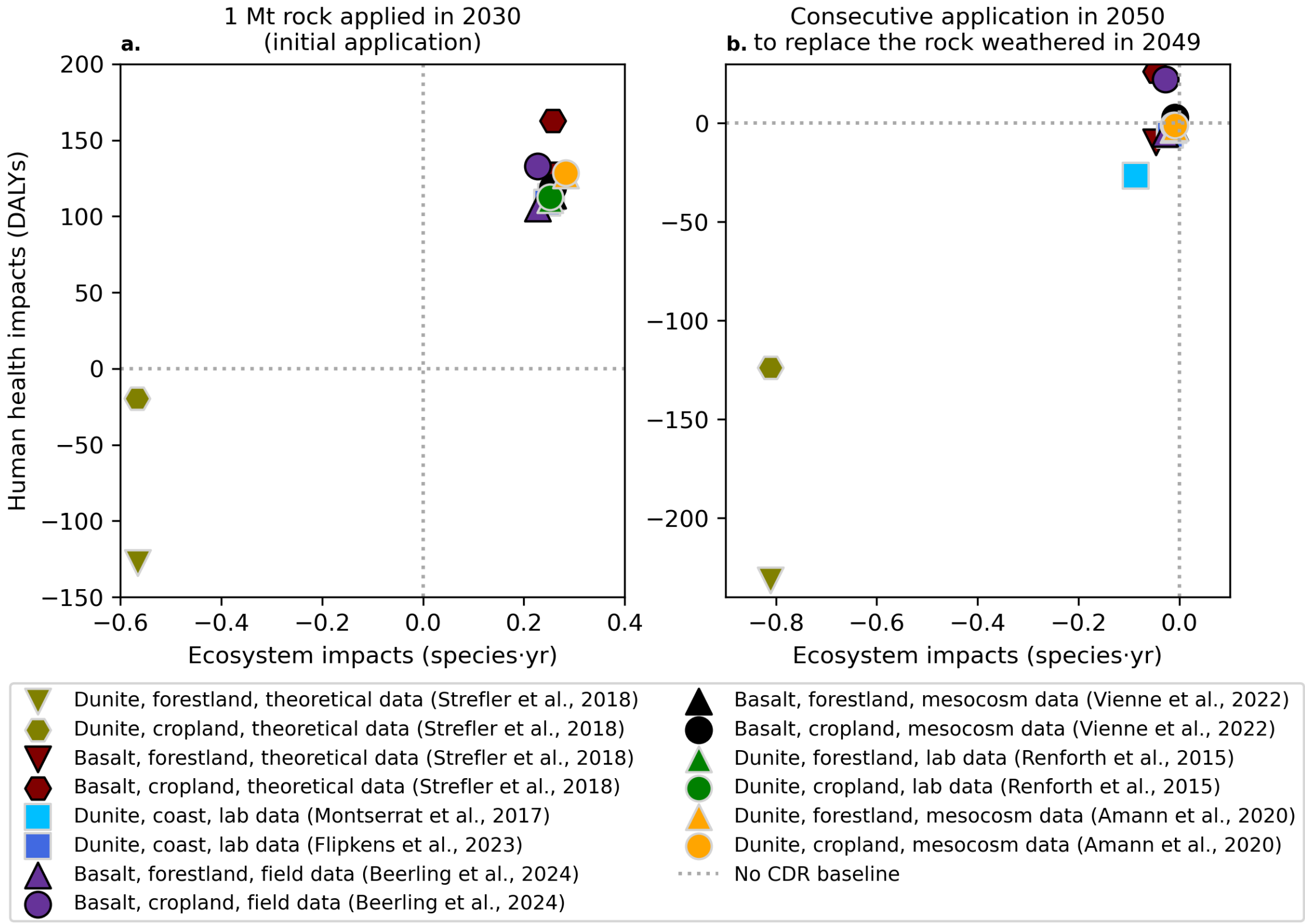

Fig. S8. Human health and ecosystem impacts of applying 1 Mt of rock particles in 2030, and the amount of rock weathered the previous year in 2050, considering experimental[18–23] and theoretical[15] data for the application of dunite and basalt on coastal and inland (forestlands and croplands) environments. **a** Rock application in 2030 for the first time. **b** Consecutive rock application in 2050.

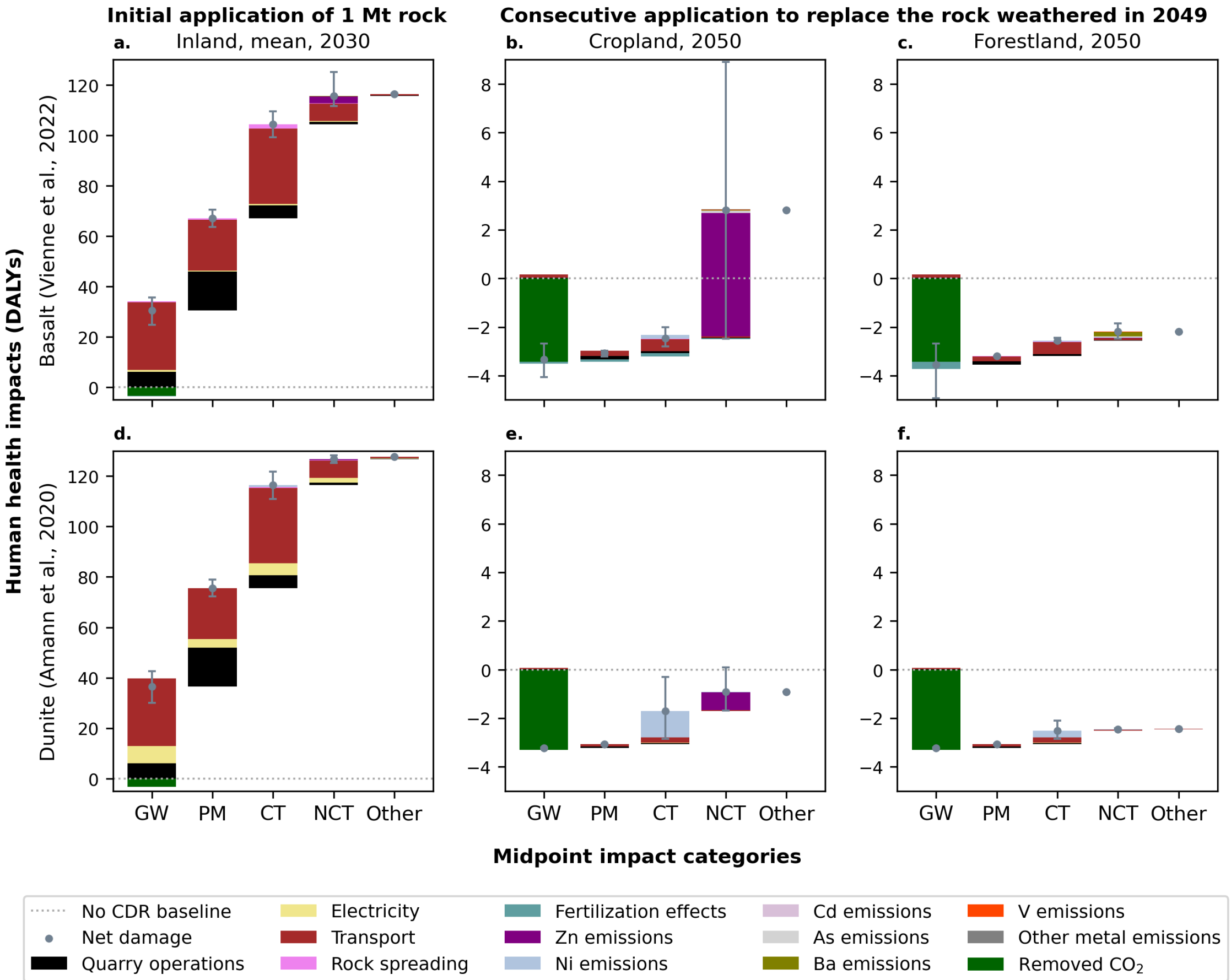

Fig. S9. Human health impacts of applying 1 Mt of rock particles in 2030 and the amount of rock weathered the previous year in 2050, considering initial rock application in 2030 and consecutive applications in subsequent years. Data from [20,22]. Error bars represent the uncertainty in the results. Impacts disaggregated by the contribution of i) unit processes and direct emissions, and ii) environmental mechanisms: global warming (GW), fine particulate matter formation (PM), carcinogenic toxicity (CT), non-carcinogenic toxicity (NCT), and others (tropospheric ozone formation, stratospheric ozone depletion, ionizing radiation, and water consumption). **a** Basalt application to cropland/forestland (mean), 2030. **b** Basalt application to cropland, 2050. **c** Basalt application to forestland, 2050. **d** Dunite application to cropland/forestland (mean), 2030. **e** Dunite application to cropland, 2050. **f** Dunite application to forestland, 2050.

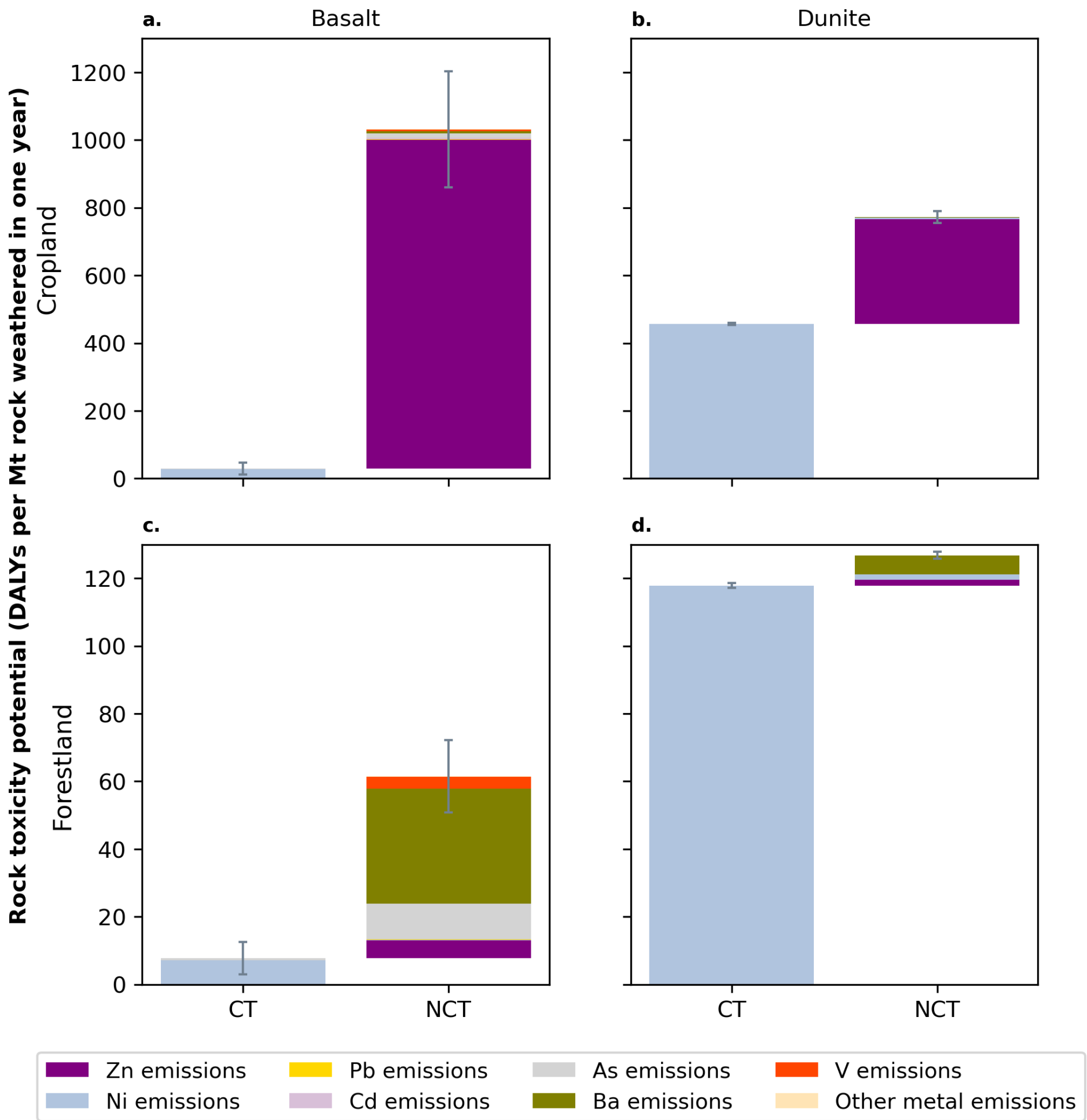

Fig. S10. Rock toxicity potential. Human toxicity impacts due to the complete weathering of 1 Mt of rock in one year, assuming 100% of metal content released into the soil. Carcinogenic (CT) and non-carcinogenic (NCT) toxicity impacts of **a** Basalt application to cropland. **b** Dunite application to cropland. **c** Basalt application to forestland. **d** Dunite application to forestland.

Figure S11 shows the probability distribution of the most impactful metal emissions, derived from the Monte Carlo simulation results. The probability distributions of zinc emissions from basalt (Figure S11a) and dunite (Figure S11c), alongside nickel emissions from dunite (Figure S11d), exhibit a uniform-like, flat-topped shape. By contrast, the probability distribution of nickel emissions from basalt (Figure S11b) features a sharp peak at lower emission values that tapers off into a long right-hand tail.

This distinct shape arises because the literature-reported concentration range for nickel in basalt (72–280 g/t rock) is significantly wider than that of zinc (Tables 1.1 and 1.2 in the Supporting Data), compounding with the other variable parameters in Equation E2 of the main manuscript during the Monte Carlo simulations to mathematically skew the resulting product distribution.

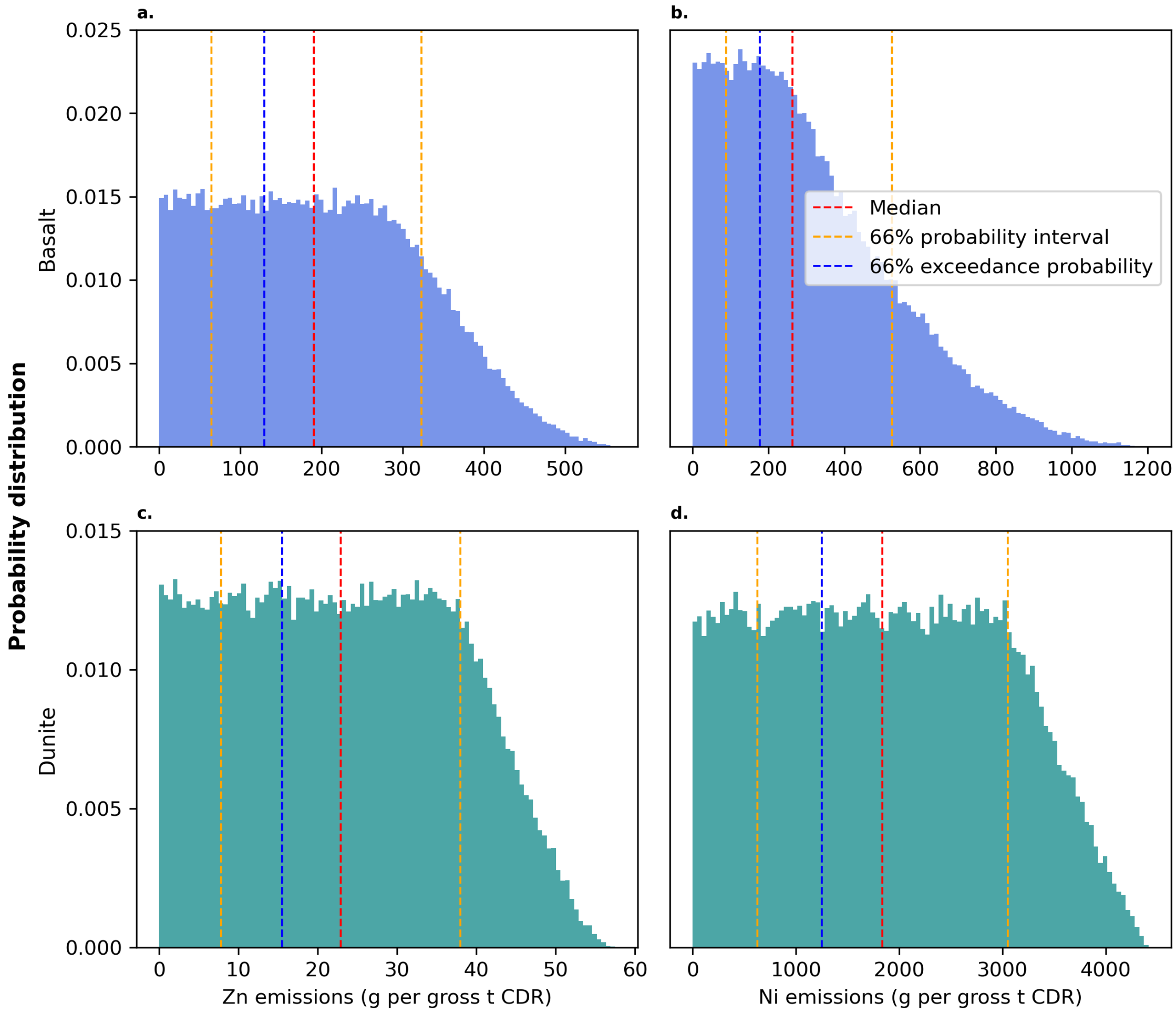

Fig. S11. Probability distributions of selected metal emissions. Data represent Monte Carlo simulation results for **a** Zinc emissions from basalt. **b** Nickel emissions from basalt. **c** Zinc emissions from dunite. **d** Nickel emissions from dunite.

Finally, Figure S12 illustrates the probability distributions of the human toxicity impacts from metal emissions, assuming basalt and dunite application to agricultural soils. The impacts are calculated with the individualistic and egalitarian perspectives of the ReCiPe 2016 method.

Notably, under the individualistic perspective–where exposure via food intake is omitted–the distributional trends observed in Figure 5 of the main manuscript are reversed. Specifically, the concurrent impacts of nickel and other metals in basalt yields a symmetric, single-peaked shape consistent with the central limit theorem[47] (Figure S12a). Conversely, dunite's toxicity impacts become predominantly driven by nickel alone, flattening its profile into a plateau-like shape in accordance with the underlying uniform probability distributions (Figure S12b). The inclusion of all exposure routes results in similarly shaped probability distributions across both the egalitarian (Figures S12c and S12d) and hierarchist (Figure 5) frameworks.

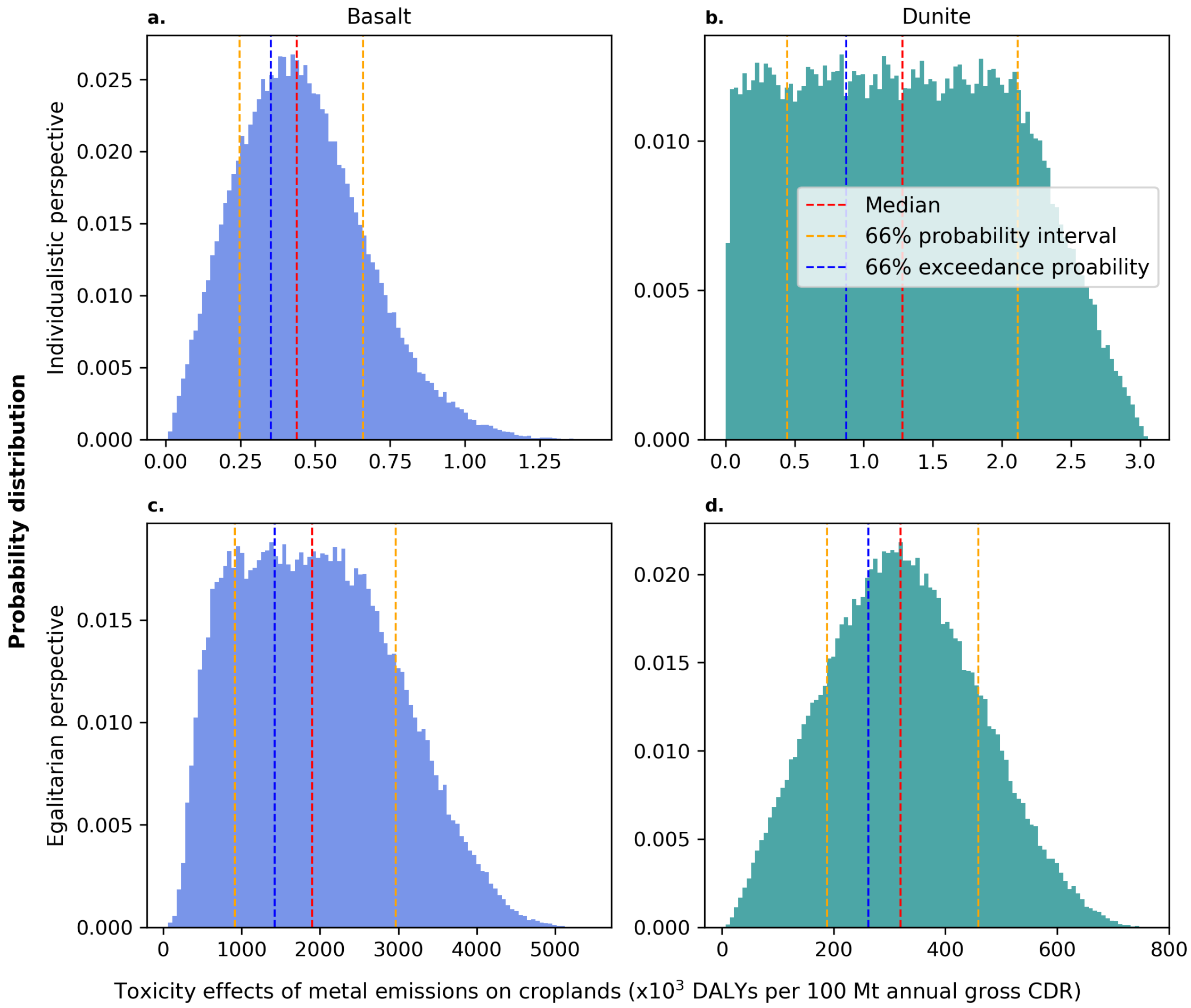

Fig. S12. Probability distributions of toxicity-driven human health impacts due to metal emissions from basalt and dunite to agricultural soil, calculated with the individualist and egalitarian perspectives of the ReCiPe 2016 method. Data represent Monte Carlo simulation results for the annual removal of 100 Mt CO_2 (gross). **a** Basalt, individualist perspective. **b** Dunite, individualist perspective. **c** Basalt, egalitarian perspective. **d** Dunite, egalitarian perspective.

5. References

(1) Levasseur, A.; Lesage, P.; Margni, M.; Deschěnes, L.; Samson, R. Considering Time in LCA: Dynamic LCA and Its Application to Global Warming Impact Assessments. *Environmental Science and Technology* **2010**, *44* (8), 3169–3174. https://doi.org/10.1021/es9030003.

(2) Smith, C.; Nicholls, Z. R. J.; Armour, K.; Collins, W.; Forster, P.; Meinshausen, M.; Palmer, M. D.; Watanabe, M. The Earth's Energy Budget, Climate Feedbacks and Climate Sensitivity-Supplementary Material. In *Climate Change 2021: The Physical Science Basis. Contribution of Working Group I to the Sixth Assessment Report of the Intergovernmental Panel on Climate Change*; 2021.

(3) Millar, J. R.; Nicholls, Z. R.; Friedlingstein, P.; Allen, M. R. A Modified Impulse-Response Representation of the Global near-Surface Air Temperature and Atmospheric Concentration Response to Carbon Dioxide Emissions. *Atmospheric Chemistry and Physics* **2017**, *17* (11), 7213–7228. https://doi.org/10.5194/acp-17-7213-2017.

(4) Huijbregts, M. A. J.; Steinmann, Z. J. N.; Elshout, P. M. F.; Stam, G.; Verones, F.; Vieira, M. D. M.; Hollander, A.; Zijp, M.; van Zelm, R. *ReCiPe 2016 v1.1. A Harmonized Life Cycle Impact Assessment Method at Midpoint and Endpoint Level. Report I: Characterization.*; National Institute for Public Health and the Environment, 2017.

(5) Van Zelm, R.; Huijbregts, M. A. J.; Van De Meent, D. USES-LCA 2.0-a Global Nested Multi-Media Fate, Exposure, and Effects Model. *International Journal of Life Cycle Assessment* **2009**, *14* (3), 282–284. https://doi.org/10.1007/s11367-009-0066-8.

(6) Rosenbaum, R. K.; Bachmann, T. M.; Gold, L. S.; Huijbregts, M. A. J.; Jolliet, O.; Juraske, R.; Koehler, A.; Larsen, H. F.; MacLeod, M.; Margni, M.; McKone, T. E.; Payet, J.; Schuhmacher, M.; Van De Meent, D.; Hauschild, M. Z. USEtox—the UNEP-SETAC Toxicity Model: Recommended Characterisation Factors for Human Toxicity and Freshwater Ecotoxicity in Life Cycle Impact Assessment. *Int J Life Cycle Assess* **2008**, *13* (7), 532–546. https://doi.org/10.1007/s11367-008-0038-4.

(7) Huijbregts, M. A. J.; Struijs, J.; Goedkoop, M.; Heijungs, R.; Jan Hendriks, A.; Van De Meent, D. Human Population Intake Fractions and Environmental Fate Factors of Toxic Pollutants in Life Cycle Impact Assessment. *Chemosphere* **2005**, *61* (10), 1495–1504. https://doi.org/10.1016/j.chemosphere.2005.04.046.

(8) Van Zelm, R.; Stam, G.; Huijbregts, M. A. J.; Van De Meent, D. Making Fate and Exposure Models for Freshwater Ecotoxicity in Life Cycle Assessment Suitable for Organic Acids and Bases. *Chemosphere* **2013**, *90* (2), 312–317. https://doi.org/10.1016/j.chemosphere.2012.07.014.

(9) Hartmann, J.; Moosdorf, N.; Lauerwald, R.; Hinderer, M.; West, A. J. Global Chemical Weathering and Associated P-Release — The Role of Lithology, Temperature and Soil Properties. *Chemical Geology* **2014**, *363*, 145–163. https://doi.org/10.1016/j.chemgeo.2013.10.025.

(10) Rimstidt, J. D.; Brantley, S. L.; Olsen, A. A. Systematic Review of Forsterite Dissolution Rate Data. *Geochimica et Cosmochimica Acta* **2012**, *99*, 159–178. https://doi.org/10.1016/j.gca.2012.09.019.

(11) Cobo, S. Enhanced Weathering Datasets, 2026. https://doi.org/10.5281/zenodo.16094445.

(12) Hangx, S. J. T.; Spiers, C. J. Coastal Spreading of Olivine to Control Atmospheric CO2 Concentrations: A Critical Analysis of Viability. *International Journal of Greenhouse Gas Control* **2009**, *3* (6), 757–767. https://doi.org/10.1016/j.ijggc.2009.07.001.

(13) Renforth, P. The Negative Emission Potential of Alkaline Materials. *Nature Communications* **2019**, *10* (1), 1401. https://doi.org/10.1038/s41467-019-09475-5.

(14) Foteinis, S.; Campbell, J. S.; Renforth, P. Life Cycle Assessment of Coastal Enhanced Weathering for Carbon Dioxide Removal from Air. *Environmental Science and Technology* **2023**, *57* (15), 6169–6178. https://doi.org/10.1021/acs.est.2c08633.

(15) Strefler, J.; Amann, T.; Bauer, N.; Kriegler, E.; Hartmann, J. Potential and Costs of Carbon Dioxide Removal by Enhanced Weathering of Rocks. *Environmental Research Letters* **2018**, *13* (3), 034010. https://doi.org/10.1088/1748-9326/aaa9c4.

(16) Rinder, T.; Von Hagke, C. The Influence of Particle Size on the Potential of Enhanced Basalt Weathering for Carbon Dioxide Removal - Insights from a Regional Assessment. *Journal of Cleaner Production* **2021**, *315*, 128178. https://doi.org/10.1016/j.jclepro.2021.128178.

(17) Jerden, J.; Mejbel, M.; Filho, A. N. Z.; Carroll, M.; Campe, J. The Impact of Geochemical and Life-Cycle Variables on Carbon Dioxide Removal by Enhanced Rock Weathering: Development and Application of the Stella ERW Model. *Applied Geochemistry* **2024**, *167*, 106002. https://doi.org/10.1016/j.apgeochem.2024.106002.

(18) Montserrat, F.; Renforth, P.; Hartmann, J.; Leermakers, M.; Knops, P.; Meysman, F. J. R. Olivine Dissolution in Seawater: Implications for CO2 Sequestration through Enhanced Weathering in Coastal Environments. *Environmental Science and Technology* **2017**, *51* (7), 3960–3972. https://doi.org/10.1021/acs.est.6b05942.

(19) Flipkens, G.; Fuhr, M.; Fiers, G.; Meysman, F. J. R.; Town, R. M.; Blust, R. Enhanced Olivine Dissolution in Seawater through Continuous Grain Collisions. *Geochimica et Cosmochimica Acta* **2023**, *359*, 84–99. https://doi.org/10.1016/j.gca.2023.09.002.
(20) Amann, T.; Hartmann, J.; Struyf, E.; De Oliveira Garcia, W.; Fischer, E. K.; Janssens, I.; Meire, P.; Schoelynck, J. Enhanced Weathering and Related Element Fluxes - A Cropland Mesocosm Approach. *Biogeosciences* **2020**, *17* (1), 103–119. https://doi.org/10.5194/bg-17-103-2020.
(21) Renforth, P.; Pogge von Strandmann, P. A. E.; Henderson, G. M. The Dissolution of Olivine Added to Soil: Implications for Enhanced Weathering. *Applied Geochemistry* **2015**, *61*, 109–118. https://doi.org/10.1016/J.APGEOCHEM.2015.05.016.
(22) Vienne, A.; Poblador, S.; Portillo-Estrada, M.; Hartmann, J.; Ijiehon, S.; Wade, P.; Vicca, S. Enhanced Weathering Using Basalt Rock Powder: Carbon Sequestration, Co-Benefits and Risks in a Mesocosm Study With Solanum Tuberosum. *Front. Clim.* **2022**, *4*, 869456. https://doi.org/10.3389/fclim.2022.869456.
(23) Beerling, D. J.; Epihov, D. Z.; Kantola, I. B.; Masters, M. D.; Reershemius, T.; Planavsky, N. J.; Reinhard, C. T.; Jordan, J. S.; Thorne, S. J.; Weber, J.; Val Martin, M.; Freckleton, R. P.; Hartley, S. E.; James, R. H.; Pearce, C. R.; DeLucia, E. H.; Banwart, S. A. Enhanced Weathering in the US Corn Belt Delivers Carbon Removal with Agronomic Benefits. *Proc. Natl. Acad. Sci. U.S.A.* **2024**, *121* (9), e2319436121. https://doi.org/10.1073/pnas.2319436121.
(24) Lewis, A. L.; Sarkar, B.; Wade, P.; Kemp, S. J.; Hodson, M. E.; Taylor, L. L.; Yeong, K. L.; Davies, K.; Nelson, P. N.; Bird, M. I.; Kantola, I. B.; Masters, M. D.; DeLucia, E.; Leake, J. R.; Banwart, S. A.; Beerling, D. J. Effects of Mineralogy, Chemistry and Physical Properties of Basalts on Carbon Capture Potential and Plant-Nutrient Element Release via Enhanced Weathering. *Applied Geochemistry* **2021**, *132*, 105023. https://doi.org/10.1016/j.apgeochem.2021.105023.
(25) Kantola, I. B.; Masters, M. D.; Blanc-Betes, E.; Gomez-Casanovas, N.; DeLucia, E. H. Long-term Yields in Annual and Perennial Bioenergy Crops in the Midwestern United States. *GCB Bioenergy* **2022**, *14* (6), 694–706. https://doi.org/10.1111/gcbb.12940.
(26) Wu, W.; Qu, S.; Nel, W.; Ji, J. The Influence of Natural Weathering on the Behavior of Heavy Metals in Small Basaltic Watersheds: A Comparative Study from Different Regions in China. *Chemosphere* **2021**, *262*, 127897. https://doi.org/10.1016/j.chemosphere.2020.127897.
(27) Madankan, M.; Kantzas, E. P.; Espinosa, R. M. E.; Vetter, S. H.; Koh, L.; Smith, P.; Beerling, D. J.; Renforth, P. Larger Rock Extraction Sites Could Improve the Efficiency of Enhanced Rock Weathering in the United Kingdom. *Commun Earth Environ* **2025**, *6* (1), 666. https://doi.org/10.1038/s43247-025-02656-9.
(28) Pehl, M.; Schreyer, F.; Luderer, G. Modelling Long-Term Industry Energy Demand and CO_2 Emissions in the System Context Using REMIND (Version 3.1.0). *Geoscientific Model Development* **2024**, *17* (5), 2015–2038. https://doi.org/10.5194/gmd-17-2015-2024.
(29) Riahi, K.; van Vuuren, D. P.; Kriegler, E.; Edmonds, J.; O'Neill, B. C.; Fujimori, S.; Bauer, N.; Calvin, K.; Dellink, R.; Fricko, O.; Lutz, W.; Popp, A.; Cuaresma, J. C.; KC, S.; Leimbach, M.; Jiang, L.; Kram, T.; Rao, S.; Emmerling, J.; Ebi, K.; Hasegawa, T.; Havlik, P.; Humpenöder, F.; Da Silva, L. A.; Smith, S.; Stehfest, E.; Bosetti, V.; Eom, J.; Gernaat, D.; Masui, T.; Rogelj, J.; Strefler, J.; Drouet, L.; Krey, V.; Luderer, G.; Harmsen, M.; Takahashi, K.; Baumstark, L.; Doelman, J. C.; Kainuma, M.; Klimont, Z.; Marangoni, G.; Lotze-Campen, H.; Obersteiner, M.; Tabeau, A.; Tavoni, M. The Shared Socioeconomic Pathways and Their Energy, Land Use, and Greenhouse Gas Emissions Implications: An Overview. *Global Environmental Change* **2017**, *42*, 153–168. https://doi.org/10.1016/J.GLOENVCHA.2016.05.009.
(30) Beerling, D. J.; Kantzas, E. P.; Lomas, M. R.; Wade, P.; Eufrasio, R. M.; Renforth, P.; Sarkar, B.; Andrews, M. G.; James, R. H.; Pearce, C. R.; Mercure, J. F.; Pollitt, H.; Holden, P. B.; Edwards, N. R.; Khanna, M.; Koh, L.; Quegan, S.; Pidgeon, N. F.; Janssens, I. A.; Hansen, J.; Banwart, S. A. Potential for Large-Scale CO2 Removal via Enhanced Rock Weathering with Croplands. *Nature* **2020**, *583* (7815), 242–248. https://doi.org/10.1038/s41586-020-2448-9.
(31) Tu, Y.; Rafols, R.; Xu, Y.; Butler, N.; Ababneh, L.; Tao, F.; Ramanathan, V.; Houlton, B. Z.; Liao, C. Scaling up Enhanced Rock Weathering for Equitable Climate Change Mitigation. *Commun. Sustain.* **2026**, *1* (1), 32. https://doi.org/10.1038/s44458-026-00034-w.
(32) Cobo, S.; Negri, V.; Valente, A.; Reiner, D. M.; Hamelin, L.; Dowell, N. M.; Guillén-Gosálbez, G. Sustainable Scale-up of Negative Emissions Technologies and Practices: Where to Focus. *Environ. Res. Lett.* **2023**, *18* (2), 023001. https://doi.org/10.1088/1748-9326/acacb3.
(33) Anda, M. Cation Imbalance and Heavy Metal Content of Seven Indonesian Soils as Affected by Elemental Compositions of Parent Rocks. *Geoderma* **2012**, *189–190*, 388–396. https://doi.org/10.1016/j.geoderma.2012.05.009.
(34) Alloway, B. J. Heavy Metals in Soils. Trace Metals in Metalloids and Soils and Their Bioavailability. In *Environmental pollution*; Alloway, B. J., Trevor, J. T., Eds.; Springer, 2013; Vol. 22. https://doi.org/10.1007/978-94-007-4470-7.

(35) Wang, H.; Li, X.; Chen, Y.; Li, Z.; Hedding, D. W.; Nel, W.; Ji, J.; Chen, J. Geochemical Behavior and Potential Health Risk of Heavy Metals in Basalt-Derived Agricultural Soil and Crops: A Case Study from Xuyi County, Eastern China. *The Science of the total environment* **2020**, *729*, 139058. https://doi.org/10.1016/j.scitotenv.2020.139058.
(36) Buckingham, F.; Henderson, G. M.; Holdship, P.; Renforth, P. Soil Core Study Indicates Limited CO2 Removal by Enhanced Weathering in Dry Croplands in the UK. *Applied Geochemistry* **2022**, *147*, 105482. https://doi.org/10.1016/j.apgeochem.2022.105482.
(37) Kelland, M. E.; Wade, P. W.; Lewis, A. L.; Taylor, L. L.; Sarkar, B.; Andrews, M. G.; Lomas, M. R.; Cotton, T. E. A.; Kemp, S. J.; James, R. H.; Pearce, C. R.; Hartley, S. E.; Hodson, M. E.; Leake, J. R.; Banwart, S. A.; Beerling, D. J. Increased Yield and CO2 Sequestration Potential with the C4 Cereal Sorghum Bicolor Cultivated in Basaltic Rock Dust-Amended Agricultural Soil. *Global Change Biology* **2020**, *26* (6), 3658–3676. https://doi.org/10.1111/gcb.15089.
(38) Bourg, A. C. M.; Loch, J. P. G. Mobilization of Heavy Metals as Affected by pH and Redox Conditions. In *Biogeodynamics of Pollutants in Soils and Sediments*; 1995; pp 87–102. https://doi.org/10.1007/978-3-642-79418-6_4.
(39) McClain, C. N.; Maher, K. Chromium Fluxes and Speciation in Ultramafic Catchments and Global Rivers. *Chemical Geology* **2016**, *426*, 135–157. https://doi.org/10.1016/j.chemgeo.2016.01.021.
(40) Sharma, N.; Wang, Z.; Catalano, J. G.; Giammar, D. E. Dynamic Responses of Trace Metal Bioaccessibility to Fluctuating Redox Conditions in Wetland Soils and Stream Sediments. *ACS Earth Space Chem.* **2022**, *6* (5), 1331–1344. https://doi.org/10.1021/acsearthspacechem.2c00031.
(41) Hartmann, J.; West, A. J.; Renforth, P.; Köhler, P.; De La Rocha, C. L.; Wolf-Gladrow, D. A.; Dürr, H. H.; Scheffran, J. Enhanced Chemical Weathering as a Geoengineering Strategy to Reduce Atmospheric Carbon Dioxide, Supply Nutrients, and Mitigate Ocean Acidification. *Reviews of Geophysics* **2013**, *51* (2), 113–149. https://doi.org/10.1002/rog.20004.
(42) Holden, F. J.; Davies, K.; Bird, M. I.; Hume, R.; Green, H.; Beerling, D. J.; Nelson, P. N. In-Field Carbon Dioxide Removal via Weathering of Crushed Basalt Applied to Acidic Tropical Agricultural Soil. *Science of the Total Environment* **2024**, *955*, 176568. https://doi.org/10.1016/j.scitotenv.2024.176568.
(43) Xu, T.; Yuan, Z.; Vicca, S.; Goll, D. S.; Li, G.; Lin, L.; Chen, H.; Bi, B.; Chen, Q.; Li, C.; Wang, X.; Wang, C.; Hao, Z.; Fang, Y.; Beerling, D. J. Enhanced Silicate Weathering Accelerates Forest Carbon Sequestration by Stimulating the Soil Mineral Carbon Pump. *Global Change Biology* **2024**, *30* (8), 1–17. https://doi.org/10.1111/gcb.17464.
(44) Xu, T.; Li, H.; Vicca, S.; Goll, D. S.; Beerling, D. J.; Chen, Q.; Bi, B.; Yang, Z.; Wang, X.; Yuan, Z. Enhanced Rock Weathering Promotes Soil Organic Carbon Accumulation: A Global Meta-Analysis Based on Experimental Evidence. *Global Change Biology* **2025**, *31* (9), e70483. https://doi.org/10.1111/gcb.70483.
(45) Goll, D. S.; Ciais, P.; Amann, T.; Buermann, W.; Chang, J.; Eker, S.; Hartmann, J.; Janssens, I.; Li, W.; Obersteiner, M.; Penuelas, J.; Tanaka, K.; Vicca, S. Potential CO2 Removal from Enhanced Weathering by Ecosystem Responses to Powdered Rock. *Nature Geoscience* **2021**, *14* (8), 545–549. https://doi.org/10.1038/s41561-021-00798-x.
(46) Vakilifard, N.; Kantzas, E. P.; Edwards, N. R.; Holden, P. B.; Beerling, D. J. The Role of Enhanced Rock Weathering Deployment with Agriculture in Limiting Future Warming and Protecting Coral Reefs. *Environmental Research Letters* **2021**, *16* (9), 094005. https://doi.org/10.1088/1748-9326/ac1818.
(47) Fischer, H. *A History of the Central Limit Theorem: From Classical to Modern Probability Theory*; Springer: New York, NY, 2011. https://doi.org/10.1007/978-0-387-87857-7.
